# “Chemobrain” in childhood cancer survivors—the impact on social, academic, and daily living skills: a qualitative systematic review

**DOI:** 10.1007/s00520-023-07985-z

**Published:** 2023-08-22

**Authors:** Ines Semendric, Danielle Pollock, Olivia J. Haller, Rebecca P. George, Lyndsey E. Collins-Praino, Alexandra L. Whittaker

**Affiliations:** 1 School of Biomedicine, The University of Adelaide, Adelaide, South Australia Australia; 2 Health Evidence Synthesis, Recommendations and Impact (HESRI), School of Public Health, The University of Adelaide, Adelaide, South Australia Australia; 3 School of Animal and Veterinary Sciences, The University of Adelaide, Roseworthy, South Australia Australia

**Keywords:** Childhood cancer, Cognitive impairment, Chemobrain, Survivorship, Academic performance, Cancer and oncology

## Abstract

**Purpose:**

To examine children’s experiences of chemotherapy-induced cognitive impairment––colloquially “chemobrain”––and the impact on children’s social, academic, and daily living skills via a qualitative systematic review. Experiencing chemotherapy as a child, when the brain is still developing, may cause lifelong detriment to survivors’ lives. There is a significant gap in understanding their lived experience, including the self-identified barriers that children face following treatment. Such a gap can only be fully bridged by listening to the child’s own voice and/or parent proxy report through an exploration of the qualitative research literature.

**Methods:**

A search of MEDLINE, Embase, PsycINFO, and CINAHL databases was conducted. Inclusion criteria were qualitative studies with a focus on children (0–18 years) during and/or following chemotherapy treatment and explored children’s experiences of chemobrain.

**Results:**

Two synthesized findings were identified from six studies. (1) Chemobrain has an academic and psychosocial impact, which may not be understood by education providers. (2) Children and their parents have concerns about their reintegration and adaptation to school, social lives, and their future selves as independent members of society. Children’s experiences primarily related to changes in their academic and social functioning.

**Conclusion:**

This review highlights two important considerations: (1) the lived experiences of pediatric childhood cancer survivors guiding where future interventions should be targeted, and (2) a need to perform more qualitative research studies in this area, as well as to improve the quality of reporting among the existing literature, given that this is a current gap in the field.

**Supplementary Information:**

The online version contains supplementary material available at 10.1007/s00520-023-07985-z.

## Background

Each year, an estimated 400,000 children and adolescents between the ages of 0 and 19 develop cancer worldwide [1]. Of these, the most common diagnoses include leukemias, brain tumors, and other central nervous system (CNS) cancers [2]. Age is a strong influencing factor, with the highest incidence of cancer and death in children aged 1–4 years old [3]. Standard therapies include the use of chemotherapy, surgery, and/or radiotherapy [1].

Advances in cancer treatment and supportive care have resulted in vast improvements in survival rates over the past 50 years in children with a cancer diagnosis, with a current event-free 5-year relative survival rate of 85–90% [4–6]. As a result, issues of survivorship are more prominent, with approximately 60–90% of childhood cancer survivors developing one or more chronic conditions affecting cardiovascular, renal, ocular, gastrointestinal, pulmonary, endocrine, musculoskeletal, neurological, immune, psychological, and reproductive systems—many of which are disabling conditions [7]. It is therefore evident that cancer, and its treatments, are different in children compared to adults, likely due to the disruption it causes to the normal development of a child’s body and brain [8]. Of particular note is that 40–60% of pediatric cancer survivors experience significant neurocognitive deficits with respect to attention, executive functioning, processing speed, visual processing, and visual motor function [6, 9–11]. These neurocognitive deficits generally predominate when combined polychemotherapy and radiation has been used in treatment [12]. Although childhood cancer patients being treated for CNS tumors with radiotherapy, particularly whole-brain irradiation, are at the greatest risk of developing long-term neurocognitive impairments, there is evidence that patients treated with chemotherapy-only are also at significant risk [13–15]. It is important to note that cognitive impairment can also be present prior to commencing any treatments, with a diagnosis of cancer itself resulting in impaired cognition in 20–50% of patients [16–19].

The presence of cognitive impairment following chemotherapy treatment specifically is known as chemotherapy-induced cognitive impairment (CICI)––colloquially known as “chemobrain.” This debilitating condition is characterized by impairments in verbal, visual, and working memory and facets of executive functioning, such as motivation, attention, and processing speed [16, 20]. A study conducted by the Swiss Childhood Cancer Register demonstrated that childhood cancer survivors (*n* = 644) are prone to developing issues with memory, concentration, and processing speed versus their healthy siblings (*n* = 247) following cancer and its treatment(s) across diagnostic and treatment groupings [21]. The implications of these impairments typically manifest in children at school through difficulty paying attention, incomplete assignments, additional time needed to complete work, struggling to keep up with the workload, and difficulties retaining and recalling information [22]. Developmental trajectories for these children are slower than the age-expected levels, with impacts evident in their IQ and academic performance over time [22]. This is particularly concerning considering potential long-term ramifications, with many survivors of childhood cancer achieving lower levels of educational attainment, experiencing a higher risk of job discrimination, and lower levels of career success over their lifetime [23–27]. To date, clinical studies examining CICI have focused primarily on adult female breast cancer patients [20]. As such, there is an under-representation of other populations of particular concern, including children.

While the prevalence and presentation of chemobrain have been previously explored in the literature, there is currently a significant gap in understanding the lived experience of childhood cancer survivors, including the self-identified burden of treatment and barriers that children face during and following treatment. Such a gap can only be fully bridged by listening to the child’s own voice through an exploration of the qualitative research literature. Listening to the child’s own voice allows for candid insights into experiences that they deem important and the subsequent impact on their lives, prioritizing where treatment and supportive care should be implemented to achieve the greatest positive effect for childhood cancer survivors. Oftentimes, it is not plausible or ethical for children to provide self-report as they may be too young, often seen in childhood cancer populations, where the highest incidence of cancer is in those under 4 years of age, or they may be cognitively or physically impaired [3, 28]. As such, parent proxy reports may have utility for shedding light on their child’s experience when the child’s own voice was not possible to obtain. At present, the lived experience of childhood cancer survivors is underrepresented in the literature. A preliminary search yielded no systematic reviews that specifically examined the experiences of children with chemobrain, either in their own voice or through parent proxy reports. This is important, as systematic reviews are considered the most reliable sources of evidence to guide the development of clinical practice guidelines; however, traditionally, systematic reviews limit their consideration only to the quantitative literature, losing a critical component of survivors’ perceptions of impact [29, 30].

To address this gap, this review explores the experiences of pediatric populations that have received chemotherapy for cancer treatment. Specifically, the impact of chemobrain on children’s social, academic, and daily living skills is considered through the voice of the child and/or their parent. It is hoped that a greater understanding of disease burden, from a patient-centered perspective, will inform survivorship frameworks and decisions on the need for and resourcing of rehabilitation, as well as other support strategies.

## Review question

What are the experiences of children who have chemobrain, and what is the impact of chemobrain on children’s social, academic, and daily living skills as described in either their own words or through parent proxy report?

## Inclusion criteria

### Participants

Studies were eligible for inclusion if they reported on the experiences of children (0–18 years) who were receiving, or had finished, chemotherapy treatment (including those who received adjuvant therapy, such as radiotherapy) and discussed experiences of cognitive impairment, commonly known as chemobrain. Studies with partial inclusion of the age range were screened at full-text to assess whether data could be extrapolated by age. There were no restrictions on time from cessation of treatment. Participants over the age of 18 years who underwent chemotherapy as children/adolescents were not eligible to minimize recall bias [31].

### Phenomena of interest

Studies that explored the impact of chemobrain on social, academic, and/or daily living skills heard through the child’s voice, or via parent/guardian proxy reports, were eligible for inclusion. If a study presented child and parent voices, it was included if both voices were distinctly identified. If the voices were combined or not adequately identified, the study was excluded.

### Context

This review considered studies that included primary and tertiary health care settings (e.g., hospital and community-based settings), their home, and education settings. Studies were not restricted by geographic location if they were English-language publications.

### Types of studies

All peer-reviewed studies using qualitative methodology were eligible for inclusion. This included data from studies that used methods such as interviews, surveys, and focus groups. Theses and dissertations were included. Other gray literature was excluded.

## Methods

This review was conducted using JBI guidance for qualitative systematic reviews and reported according to the Preferred Reporting Items for Systematic Reviews and Meta-Analyses (PRISMA) 2020 guidelines [32, 33]. This review was registered with PROSPERO (PROSPERO Registration CRD42021240573) and conducted in accordance with an a priori protocol [34].

### Search strategy

An initial search was conducted to identify appropriate key terms, index terms, and controlled vocabulary, such as MeSH terms performed in MEDLINE via PubMed. Identified keywords included “childhood cancer survivor(s),” “pediatric,” “quality of life,” and facets of cognitive impairment such as “psychological adaptation,” “interpersonal relations,” “social skills,” and “academic performance” to broadly encompass all potential experiences. The search strategy was developed in conjunction with a medical information specialist to ensure appropriate terms and a robust search methodology. MEDLINE via Ovid, Embase, PsycINFO via Ovid, CINAHL via EBSCOhost, and ProQuest Dissertations and Theses were searched from database inception to identify studies for title and abstract screening (Online Resource 1). No date restrictions were applied to the searches. Only English-language publications were included due to a lack of ready access to translators.

### Study selection

**Fig. 1:**
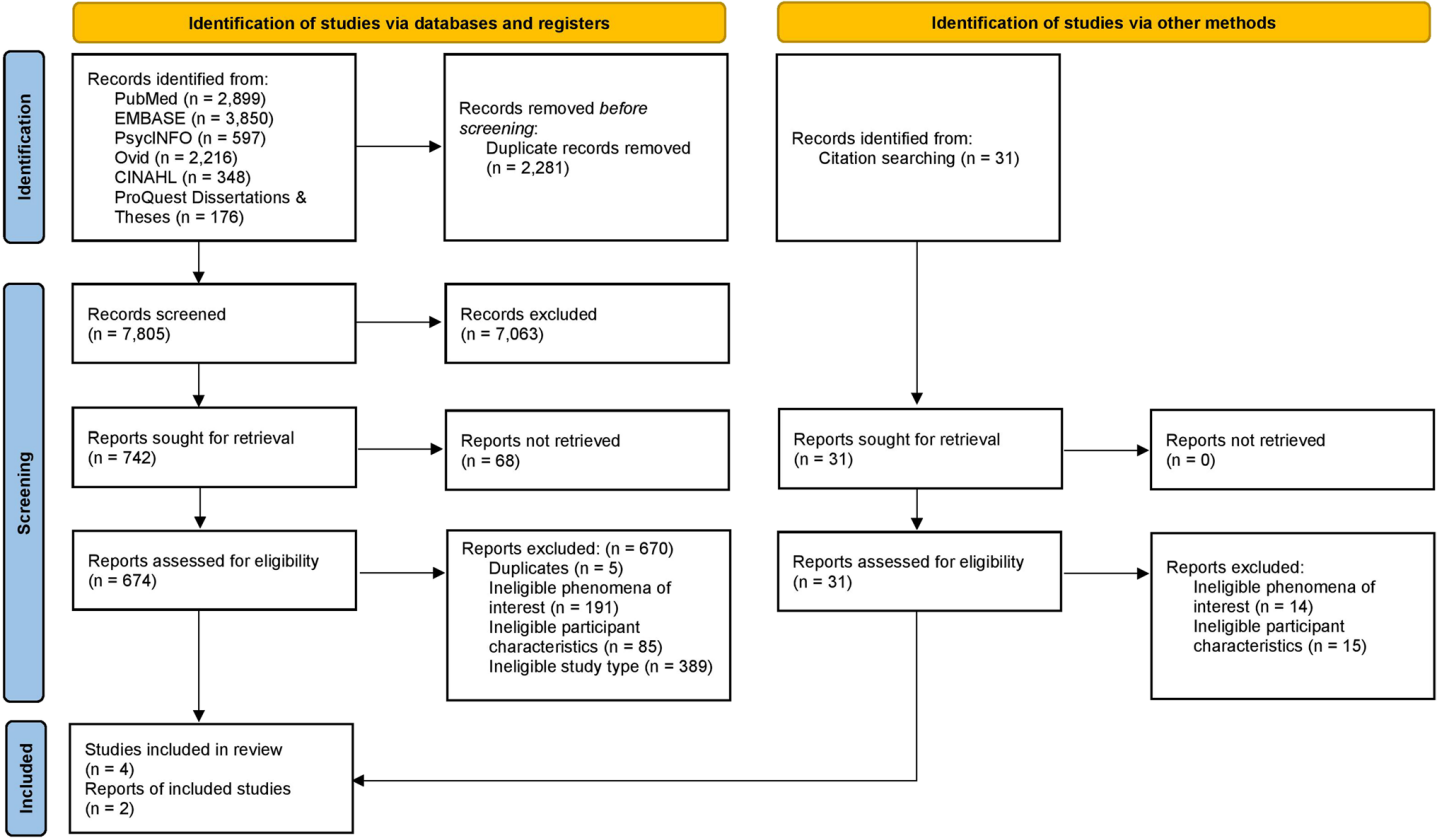
PRISMA 2020 flow diagram for new systematic reviews which included searches of databases, registers, and other sources [33]

Following searches of included databases, all citations were collated and uploaded to EndNote X9 (Clarivate Analytics, PA, USA) and imported into Covidence systematic review software (Veritas Health Innovation, Melbourne, Australia), where duplicates were removed. Where conflicts arose, a third independent reviewer was consulted (DP). The title and abstract and full-text screening process was piloted by assigning a random subset of studies (10 studies per reviewer) to two independent reviewers (IS and ALW). These reviewers assessed whether to include or exclude these studies with reasoning. To ensure consistency in the study selection, a consensus of 90% between the reviewers was attained prior to undertaking full screening. Each study was screened based on the title and abstract against the inclusion and exclusion criteria by four independent reviewers (IS, OJH, RPG, ALW). The reference lists of all included studies were screened for additional studies. Full-text and supplementary materials were imported into the JBI System for the Unified Management, Assessment, and Review of Information (JBI SUMARI; JBI, Adelaide, Australia) [35]. Full-text studies were assessed against the inclusion and exclusion criteria by four independent reviewers (IS, OJH, RPG, ALW), with a fifth reviewer being consulted where there was any disagreement (DP). Reasons for exclusion were recorded and presented. An updated search was conducted in 2022, a year from the original search. The results of both searches are reported in full, presented in a PRISMA flow diagram [33] (Fig. 1).

### Assessment of methodological quality

Studies eligible for inclusion were critically appraised for their methodological quality using the standard JBI critical appraisal checklist for qualitative research tool in JBI SUMARI [32] (Online Resource 5). Each study was appraised against 10 predefined questions for which answers “yes,” “unclear,” or “no” were applicable to determine overall quality. Two independent reviewers (IS AND ALW) conducted the critical appraisal blinded to each other’s assessment. Any conflicts that arose were resolved via discussion or consultation with a third independent reviewer (DP). All studies, regardless of the results of their methodological quality, underwent data extraction and synthesis.

### Data extraction

JBI’s standardized extraction tool was used for the six included studies (Online Resource 2) [32]. Two independent reviewers (IS and ALW) conducted data extraction. Any conflicts that arose were resolved via discussion or consultation with a third independent reviewer (DP). Extracted data included citation details, study design, country of origin, country where research was conducted, setting/context, time or time frame of data collection (if applicable), population characteristics (based on inclusion/exclusion criteria), methodology, and study outcomes.

Each finding and associated illustrations was graded for credibility (unequivocal, credible, or not supported) using the ConQual process [36]. Supplementary materials and appendices were referred to where necessary.

### Data synthesis

A meta-aggregative approach was utilized to synthesize the results in JBI SUMARI [37]. This approach involves collating similar findings to generate general statements that describe that aggregation by categorizing them based on similarity of meaning. These categories were then subjected to a synthesis to produce a single comprehensive set of synthesized findings. Only findings that achieved an unequivocal (U) or credible (C) grading were included in aggregation; unsupported findings are presented separately (Online Resource 3).

### Assessing confidence in the findings

Synthesized findings obtained from data synthesis were subject to the ConQual approach to determine confidence in the findings [36]. The Summary of Findings table including the review title, population, phenomena of interest, and context is presented. Each synthesized finding is presented, along with the evidence informing it, scores of dependability and credibility, the overall ConQual score, and relevant comments informing this process.

## Results

### Study inclusion

The original database search, and an updated search conducted a year later, identified a total of 10,117 studies. A total of 7063 studies were excluded based on title and abstract screening, with 773 remaining for full-text assessment. During the full-text screening, 699 studies were excluded due to duplicates, ineligible phenomena of interest, ineligible participant characteristics, and ineligible study type. In total, six studies were included in this review.

Details of the identification process are described in the Preferred Reporting Items for Systematic Reviews and Meta-Analyses (PRISMA 2020) flow chart [33] (Fig. 1).

### Study characteristics

For the six studies that met the eligibility criteria, publication dates ranged from 1999 to 2021. Data were provided from the USA (*n* = 2), Belgium (*n* = 1), Taiwan (*n* = 1), Canada (*n* = 1), and the UK (*n* = 1). Included studies employed semi-structured interviews (*n* = 5) and clinical interview (*n* = 1) methodology. Eighty percent of studies did not report a philosophical perspective, and 50% did not report the qualitative methodology. Both Choquette and Vance reported an interpretive philosophical perspective, while Choquette, Vance, and Chen reported descriptive methodology [38–40]. The characteristics of the included studies are reported in Online Resource 4.

### Participant characteristics

Participant characteristics from the included studies included age at diagnosis (8–16 years), age at study inclusion (7–18 years), and a range of 5.6 to 11.9 (mean) months since the cessation of treatment. Medical diagnoses included leukemias (acute lymphoblastic leukemia and acute myeloid leukemia), lymphomas (Hodgkin and non-Hodgkin), sarcoma (Ewing and osteosarcoma), testicular, and brain tumors (medulloblastoma), treated with a combination of chemotherapy, radiotherapy, bone marrow transplant, and/or surgical procedures. Seven participants attended school, with one of these participants additionally receiving special education [38–42]. One participant attended school but was previously home-schooled [43]. One participant was home-schooled but previously attended school [39]. Where specific participants were not identified, general characteristics were reported [38, 41]. Detailed characteristics of participants included in each study are reported in Table 1.X`X`XXXXXX`[Edit]

**Table 1:** Participant characteristics

Study	Participants	Sex	Age at diagnosis, years	Age at study inclusion, years	Medical diagnosis	Type of treatment	Time since cessation of treatment	Current education	Statemented (statement of special needs)
Chen et al. [40]	“Adolescent F”	Male	12.4	15	ALL	C/T + BMT	N/A	Attending school	N/A
	“Adolescent B” (parent report)	Male	13.6	18	Malignant lymphoma	C/T	N/A	Attending school	N/A
Choquette, et al. [38]	Specific participant not identified. General characteristics reported	Female (*n* = 3), male (*n* = 8)	Range 8–16	Range 13–17	Leukemia (ALL; AML) *n* = 4 Lymphomas (Hodgkin, non-Hodgkin) *n* = 2 Sarcoma (Ewing sarcoma) *n* = 2 Brain tumors *n* = 1 Others (germ cells tumors) *n* = 2	C/T with, or without, R/T	11.9 (mean months)	Attending school	N/A
Suntup [43]	Peter	Male	N/A	7	ALL	C/T	N/A	Home-schooled from kindergarten and the first 2 months of first grade Currently attending school	N/A
Vanclooster et al. [41]	“Case 3” (parent report)	Male	N/A	10	Medulloblastoma	Surgery, R/T, C/T	N/A	Attending school	N/A
Walker et al. [42]	Specific participant not identified. General characteristics reported	Female (*n* = 11), male (*n* = 18)		Mean 15.5	Hodgkin lymphoma *n* = 10 Bone tumors (Ewing sarcoma and osteosarcoma) *n* = 7 ALL *n* = 6 AML *n* = 2 Non-Hodgkin lymphoma *n* = 2 Testicular *n* = 2	C/T with, or without, R/T	5.6 (mean months)	Attending school	N/A
Vance et al. 2004 [39]	Rebecca	Female	10.22	15	Medulloblastoma	C/T, R/T	N/A	Attending school and special needs unit	Y
	Hannah	Female	9.18	18	Medulloblastoma	C/T, R/T	N/A	Attending school	N (tried but were not successful)
	Lynne	Female	9.51	18	Medulloblastoma	C/T, R/T	N/A	Home-schooled	N

*C/T* chemotherapy, *R/T* radiotherapy, *BMT* bone marrow transplant, *ALL* acute lymphoblastic leukemia, *AML* acute myelogenous leukemia, *N/A* not applicable as information was not reported.

### Critical appraisal results

Each study (*n* = 6) was critically appraised for methodological quality using the standard JBI critical appraisal checklist for qualitative research tool in JBI SUMARI [37] (Online Resource 5). Of the studies, half had issues with congruity with the research methodology, research question/objective, methods used to collect data, representation and analysis of data, and the interpretation of the results. Four studies had issues with congruity between the stated philosophical perspective and research methodology and had no statement locating the researcher culturally or theoretically. One study did not adequately represent the participant’s voice(s). All studies reported ethics approval and presented logical conclusions drawn from the analysis, or interpretation, of their data (Online Resource 5).

### Findings of the review

The findings of this review and meta-aggregation are reported in full in Online Resource 6 and 7. The final synthesized findings were graded according to the ConQual Summary of Findings, as shown in Table 2.

**Table 2:** ConQual summary of findings

“Chemobrain” in childhood cancer survivors—the impact on social, academic, and daily living skills: a qualitative systematic review Population: children (0–18 years) who were receiving chemotherapy or had finished chemotherapy treatment (including those who received adjuvant therapy such as radiotherapy alongside chemotherapy) and had experienced cognitive impairment Phenomena of interest: studies that explored the impact of chemobrain on social, academic, and/or daily living skills heard through the child’s voice, i.e., self-report or via parent/guardian proxy reports Context: target population in primary and tertiary health care settings (e.g., hospital and community-based settings including the home)				
Synthesized finding	Type of research	Dependability	Credibility	ConQual score
Synthesized finding 1: Chemobrain has an academic and psychosocial impact which may not be understood by education providers Children’s experiences with “chemobrain” manifest as problems with memory, attention, comprehension, and feeling “mentally slow” with an emphasis on functioning in the school setting. Children found it difficult to transition back to school and struggled with academic performance. Children were acutely aware of their reduced abilities and compared themselves to peers who were performing well. This comparison led to mental distress. Only one child was able to get “statemented” (given a statement of special needs) to be able to access support for their struggles. As such, resources appear to be lacking with minimal support or education provided to aid struggling children	Qualitative	Dependability: low *Comments:* The dependability score moved down two levels as the following were not addressed across the included studies: • Congruity between the research methodology and the research question • Congruity between the research methodology and the methods used • Congruity between the research methodology and representation and analysis of data • Statement locating the researcher culturally or theoretically • Statement about the influence of the researcher on the research, and vice-versa	Downgrade one level (− 1) *Comments:* The credibility score was downgraded one level as there was a mix of credible and unequivocal findings including 5 unequivocal findings and 3 credible findings	Very low
Synthesized finding 2: Children and adolescents and their parents have concerns about their reintegration and adaptation to school, social life, and their future selves as independent members of society Parents express great concern regarding their child’s future, specifically their level of independence and ability to self-care. While some children recognize and accept the changes to their abilities and adapt by reducing their workload or finding other ways to excel non-academically, other children can develop debilitating issues like social phobia without the necessary support. Additionally, many children struggle with fundamental daily living skills, such as handling money and bills, posing a significant barrier to their future employability, livelihood, and quality of life	Qualitative	Dependability: moderate *Comments:* The dependability score moved down one level as the following were not addressed across the included studies: • Congruity between the research methodology and the research question • Congruity between the research methodology and the methods used • Congruity between the research methodology and representation and analysis of data • Statement locating the researcher culturally or theoretically • Statement about the influence of the researcher on the research, and vice-versa	Downgrade one level (− 1) *Comments:* The credibility score was downgraded one level as there was a mix of credible and unequivocal findings including 7 unequivocal findings and 1 credible finding	Low

### Synthesized finding 1: Chemobrain has an academic and psychosocial impact which may not be understood by education providers

Children’s experiences with chemobrain manifest as problems with memory, attention, comprehension, and feeling “mentally slow” with an emphasis on functioning in the school setting [38–40, 42, 43]. Children found it difficult to transition back to school and struggled with academic performance [40, 42]. Children were acutely aware of their reduced abilities and compared themselves to peers who were performing well, causing mental distress [39, 41]. Only one child was able to get “statemented” (given a statement of special needs) to be able to access support [43]. As such, resources appear to be lacking, with minimal support or education provided to aid struggling children (Table 3).

**Table 3:** Detailed summary of synthesized findings

Synthesized findings	Categories	Description	Illustration
Synthesized finding 1: Chemobrain has an academic and psychosocial impact which may not be understood by education providers	Adolescent’s transition to school was impacted	Children found it difficult to transition back to school and struggled with academic performance as chemobrain affected their ability to get back into school and catch up on schoolwork [38–42]	“Like the chemo brain and stuff this year, it got hard, harder for me to process things in school.” (Walker et al., 2019, p.17)
	Provision of support and/or communication	One parent reported their child being “statemented” (being given a statement of special needs) [39]. As a result, the child received extra attention and help at school to accommodate for their difficulties	“She (Rebecca) was supposed to get a lot of extra help. In a way it’s like a special school within a mainstream school, a special unit” (Vance, Eiser and Horne, 2004, p.276)
	Academic performance	One child found it difficult to understand school content and what their teacher was teaching, they worried about their school performance [41]. Parents noted that while their child’s grades didn’t appear to suffer, he became slow to complete work, would daydream and become distracted while doing homework, and exhibit high energy levels that prevented him from focusing on his work [40]	“I didn’t understand what the teachers were teaching [shrugged his shoulders]. So I worried about my poor school performance.” (Chen et al., 2015, p.223)
	Feeling different	One child was acutely aware of their reduced abilities and compared themselves to their peers who were performing well [41]	“He knows that he lacks certain skills, now even more than before. (Case 3, parent)” (Vanclooster et al., 2021, p.2615)
	Attention, concentration, and memory	Children noted that the mental aspect was difficult with the feeling that they were slow[38]. Additionally, parents reported their child displayed behaviors that are indicative of attention difficulties, such as fidgeting, difficulty following instructions, difficulty sustaining attention, and hyperactivity [43]	“It’s just the mental stuff. Like that’s the main part… It’s just…I’m slow.” (Choquette 2016, p.399)
Synthesized finding 2: Children and adolescents and their parents have concerns about their reintegration and adaption to school, social life, and their future selves as independent members of society	Independence and ability to self-care	Parents expressed significant concern for their child’s ability to be independent and self-care [39]. Parents worried about leaving their child at home alone and their child’s ability to be fully independent and achieve daily living activities [39]	“I’ve been really worried about leaving her in the house, but I’ve had to, and I have to keep telling myself that she’s 15 now and that sometimes I do have to go out (Rebecca).” (Vance, Eiser and Horne, 2004, p.281)
	Social reintegration and understanding the experience and change they have gone through	One child developed social phobia as a result of the academic problems she suffered [39]. She excelled academically pre-illness but struggled to maintain her achievements when she returned to mainstream school. Her mother tried for her to be “statemented” (given a statement of special needs) but was not approved	“She would genuinely have been feeling sick, because she was frightened of going to school”—Hannah's mother (Vance, Eiser and Horne, 2004, p.276)
	Concern for future	Parents had significant concern for their child’s future, particularly pertaining to their ability to provide for themselves and live independently [39]. They are apprehensive about their child being able to obtain and maintain employment and provide for themselves financially without support [39]	“I mean I think she’s going to need a lot of supervision and mentoring, whatever she does […] She won’t be employable unless you’ve got a very considerate employer” (Vance, Eiser and Horne, 2004, p.277)
	Adaption to a new way of being	Children recognized that who they are, and their lifestyle, in survivorship is different. Some children are accepting these changes and accommodating their lifestyle to follow suit, such as dropping physical extracurricular activities and advanced academic classes, or developing new ways to excel that aren’t academic (e.g., music) [39, 42]. Another child found it difficult to discern what behavior is related to “chemobrain” and what behavior is related to “just being a teenager” and finds this can cause some conflict between his mother and himself [42]	“My parents want me to like, do like running start in junior year and like do honors classes. But I like, I wanna do that too so I can like excel, but I know like I can’t push myself too much, but I also like, "cause like the chemo brain kind of isn’t, doesn’t wanna like work with that.” (Walker et al., 2019, p.17)

### Synthesized finding 2: Children and adolescents and their parents have concerns about their reintegration and adaption to school, social life, and their future selves as independent members of society

Parents express concern regarding their child’s future, specifically their level of independence and ability to self-care [39]. While some children recognize and accept the changes to their abilities and adapt by reducing their workload or finding other ways to excel non-academically, other children can develop debilitating issues like social phobia without the necessary support [39, 42]. Many children additionally struggle with fundamental daily living skills such as hygiene, feeding themselves, and handling money, posing a significant barrier to their future employability, livelihood, and quality of life [39] (Table 3).

## Discussion

To our knowledge, this is the first systematic review of qualitative evidence exploring how chemobrain affects a child’s social, academic, and daily living skills from the perspective of the child and/or parent proxy report. Our primary finding is that children who have had childhood cancer, and have subsequently suffered cognitive changes (chemobrain), find their lives impacted, on a personal level, interpersonal level, and academic level. Their parents express concern for their future as a result.

Chemobrain affected children’s memory, concentration, attention, and learning, resulting in poor academic performance. Some children demonstrated more specific attentional deficits. Children found it difficult to get back to school and catch up on missed work, could not remember school material, could not understand the teacher, felt mentally slow, took a long time to complete work, and had poor performance in tests. Changes in their functioning made children feel different from their peers, who could keep up with the workload or extracurricular activities. Indeed, the presentation of these symptoms is consistent with what has been previously demonstrated in this population with quantitative research studies highlighting that childhood cancer survivors struggle with memory, concentration, and attention interfering with their schooling, as demonstrated by lower academic achievement, incomplete assignments, struggling to keep up with the workload, and difficulties in retaining and recalling information they learn [21, 22]. Long-term, these issues manifest as lower levels of educational attainment, higher risk of job discrimination, and lower levels of career success over their lifetime [23–27].

Despite evidence of these challenges voiced by the children in the included studies, it is difficult to tease apart whether these difficulties are due to chemobrain specifically or other factors associated with treatment. Time spent away from school due to health may result in significant impacts on a child’s academic performance and abilities upon returning to school following treatment. Children who miss school for prolonged periods of time due to chronic health issues are three times more likely to have developmental and behavioral conditions than their peers [44]. Additionally, studies have demonstrated that children who miss more days of school report lower academic performance and achievement [45]. Conversely, the physical side effects of cancer and its treatment can also impact cognitive performance, particularly fatigue. Cancer-related fatigue (CRF), in particular, is a condition associated with diminished concentration, attention, motivation, and work-related cognitive limitations in adult breast cancer patients and is associated with decreased quality of life long-term [46, 47].

In addition to physical and cognitive ramifications of cancer and its treatment, psychological effects are also more prevalent in this population, with symptoms of anxiety, depression, post-traumatic stress disorder, and low self-esteem potentially exacerbating other negative experiences, such as poor performance at school, difficulty attaining employment, and deficits in socializing [24, 25, 27]. Evident in this review, some children would compare their own performance to how well their peers were doing and would feel upset. Children were confronted with these changes upon returning to school. Alongside academic changes, children’s social landscape also changed, with some children withdrawing. In contrast, some children adapted to their new way of being and confronted these changes. One child took up art as an alternative way to excel at school without excelling in academic performance. Children who recognized that their abilities and lifestyles in survivorship are different and found acceptance of these changes accommodated their lifestyle to follow suit. The potential presentation of psychological effects may put some children at more risk than others of experiencing a lower quality of life post-cancer and its treatment [24, 25, 27]. Importantly, all these factors may be occurring in conjunction, worsening the personal burden and impact on short-term and long-term quality of life.

Parents recognized the impact that these changes had on their child’s academic performance. Many parents sought help for their child, primarily through the form of being statemented (given a statement of special needs). Although parents’ experiences were not the focus of this review, a key theme related to the ability to access support. Many parents found that obtaining support for their children was a difficult process and they had to fight for help as monitoring for their child’s cancer diagnosis had ceased years prior. As a result, the education departments deemed there was no evidence of needed support. Many parents failed to obtain the help needed from the education department, leaving these children in a vulnerable position. Resources available for parents/caregivers appeared to be lacking, with minimal support or education provided to the education department, schools, or parents to navigate the process for obtaining additional support or where to obtain it. As such, a secondary finding demonstrates that there is a lack of education support and resources from educational departments for these children and their caregivers. No mention was made as to whether these children were still attending hospitals/clinics in an outpatient capacity, or if they were attending survivorship organizations for additional support.

Additionally, parents expressed significant concern about their child’s ability to be independent and how this may impact their child’s future. Indeed, it is estimated that 60% of childhood CNS tumor survivors do not reach full independence in relation to factors such as employment, living independently, taking care of themselves, obtaining a driver’s license, and marital status [48]. The risk of lower levels of independence was associated with craniospinal irradiation treatment and younger age at diagnosis, putting childhood cancer survivors at particular risk, with the highest incidence of cancer in children aged 1–4 years old [3, 48].

While the experiences children shared primarily related to their academic functioning and social functioning, given how school and friends are critical factors for them during this period of their lives, parents, instead, showed a significant concern for their child’s future—particularly in their ability to be independent and self-care. Despite the evident impact and concern for future outcomes, adequate support and resources were not made available to improve short-term and long-term outcomes for these children.

Despite the broad inclusion criteria to cover those who were undergoing treatment or had completed treatment, all included studies were of children who had completed treatment, likely due to the inherent ethical risks of children undergoing treatment. Additionally, this review had as an inclusion criterion that cancer treatment had to include chemotherapeutic agents, with or without adjuvant therapies, but did not consider treatment strategies without the use of chemotherapy. While individual diagnoses and treatment(s) will vary greatly between patients, it is well established that children being treated for central nervous system (CNS) tumors with radiation therapy, particularly whole-brain irradiation, are at the greatest risk of developing long-term neurocognitive impairments [49]. From a patient perspective, it is of little consequence whether the cognitive decline results from chemotherapy, or some other treatment, the cancer itself, or as a result of missed schooling; the experience of life impact is just as valid. As such, the external validity of the study remains sound. However, more evidence may have been sourced by the inclusion of all cancer types and treatments, regardless of whether adjuvant chemotherapy was administered or not. Additionally, there are few qualitative study designs that investigate this topic utilizing the terminology of “chemobrain,” “chemotherapy-induced cognitive impairment,” or more broadly “cancer-related cognitive impairment,” leading to the inclusion of only six relevant studies that addressed the criteria for these conditions. Rather than specific focuses on outcomes of chemobrain, such as memory or attention, there is a greater focus on how general changes integrate with the children’s lives, making it difficult to discern what outcomes are the result of physical changes, fatigue, time spent at the hospital, memory issues, and so forth. As such, this review was supported by few studies, i.e., limited evidence, however, does identify an important research gap. Within the included studies, as reflected by the quality appraisal, there was a distinct lack of concise and consistent reporting regarding research paradigms and research methodology. Of the excluded studies, many public health researchers, in particular, did not report imperative methodological information, such as participant characteristics, leading to study exclusion and potentially missing out on informative perspectives.

This is reflected as out of 674 studies screened for full-text, only 6 studies were eligible for inclusion. While we acknowledge that this is a small number of studies, it does not limit the value of the included studies to inform our understanding of the self-perceived impact of chemobrain on childhood cancer survivors. Furthermore, it highlights an important gap that should be communicated more widely to the field about the relatively small amount of literature currently available on the patient perspective and identifies a critical area of unmet need that should inform future research.

### Clinical implications

From the perspective of childhood cancer survivors, there is a significant area of unmet need surrounding resources, support, and education available for the child themselves, as well as their parents, and those in support positions, such as allied health and educational departments, who may be able to provide support. The use of organized educational programs directed at teachers and class peers, providing guidance on how to manage problems that are associated with school reentry for survivors, has been shown to be successful [50]. Programs provided typically cover tutoring, communication between the family, hospital, and school, education for parents/children regarding return to school, school staff training, individual education plans (IEP), neuropsychological evaluation, cognitive remediation, buddy programs, social skills programs, and planned learning support meetings [50–54]. However, the implementation of school-based interventions appears to be mainly done when legislation requires it [50]. This is particularly evident when considering the geographical location of survivors, with school reentry programs being widely implemented in North America due to legislation which protects the educational and employment rights of individuals with disabilities; however, the form varies widely across the USA due to differences in state laws [49, 50]. While some countries, such as Australia, have both the existence of legislation to protect these children, namely the *Disability Discrimination Act 1992* and *Disability Standards for Education 2005*, and available documentation on return to school, this population is still not adequately supported [55–58]. Variations and ambiguity in the existing documentation appear to lead to inconsistent care and often fail to facilitate communication between the hospital, home, and school to guide this process [57]. Australian parents report that little or no structured support is provided, with the most common form being “general classroom support” and “school counsellor,” and that accessing support was dependent on the parent’s will rather than a systemically employed approach [56]. Similarly in Europe, rather than the systemic implementation of universal guidelines, survivorship surveillance and care are being driven by research organizations, such as PanCare (Europe) and the European Society for Paediatric Oncology (SIOP). These organizations, in collaboration with 17 partners across Europe, aim to implement digital “Survivorship Passports” that provide childhood/adolescent cancer survivors with custom survivorship plans based on individual medical history [59, 60]. While a promising initiative, these approaches still rely on the survivor and their caregivers to drive their own survivorship care implementation. Interestingly, there is a lack of concordance between government initiatives and these organizations. The European Union has launched the “EU4Health Programme” from 2021 to 2027 utilizing a similar design, the “Cancer Survivor Smart-Card,” to support childhood cancer survivors, rather than working with existing organizations to harmonize the development and implementation of such interventions [61].

Given this, there is a fundamental need for governing bodies to recognize this population and employ both supportive legislation to ensure they are protected and provided with universal resources by their primary healthcare providers following remission with a significant need to focus on harmonizing guidelines and implementation. The use of an assigned liaison for each survivor to help with the transition from hospital to school reentry may be beneficial to act as an advocate for children to access neuropsychological evaluation and occupational therapy, and be an information provider and communicator for parents and involved educational departments ensuring equitable care [50, 54].

## Conclusion

To our knowledge, this is the first systematic review of qualitative evidence to examine how chemobrain affects a child’s social, academic, and daily living skills through the voice of the children themselves and/or parent proxy report. This review highlights two important considerations. Firstly, the lived experiences of pediatric childhood cancer survivors, guiding where future interventions should be targeted. While the prevalence and presentation of chemobrain have been previously explored in the literature, we now have evidence that this is reflected in their lived experience and that these findings have a discernible impact on quality of life. Secondly, there is a need to perform more qualitative research studies in this area, as well as to improve the quality of reporting among the existing literature, given that this is a current gap in the field.

## Supplementary Information

Below is the link to the electronic supplementary material.

Supplementary file1 (PDF 270 KB)

Supplementary file2 (PDF 65 KB)

Supplementary file3 (PDF 125 KB)

Supplementary file4 (PDF 200 KB)

Supplementary file5 (PDF 170 KB)

Supplementary file6 (PDF 146 KB)

Supplementary file7 (PDF 151 KB)

## Data Availability

All data generated and/or analyzed during this study are included in the published article and its supplementary information files. Any queries may be directed to the corresponding author.
