# Supplementary material for: “Chemobrain” in childhood cancer survivors—the impact on social, academic, and daily living skills: a qualitative systematic review
Source: Support Care Cancer. 2023 Aug 22;31(9):532. doi: 10.1007/s00520-023-07985-z (PMC10444646; doi:10.1007/s00520-023-07985-z)
Supplement: Supplementary file 1 — Supplementary file1 (PDF 270 KB) [file 520_2023_7985_MOESM1_ESM.pdf]

“Chemobrain” in childhood cancer survivors – the impact on social, academic, and daily living skills: a qualitative systematic review

Ines Semendric[1*], Danielle Pollock[2], Olivia J Haller[1], Rebecca P George[1], Lyndsey E. Collins-Praino[1], Alexandra Whittaker[3]

1. School of Biomedicine, The University of Adelaide, Adelaide, South Australia

2. JBI, Faculty of Health and Medical Sciences, Adelaide, South Australia

3. School of Animal and Veterinary Sciences, The University of Adelaide, Roseworthy, South Australia

*Corresponding author: Ines Semendric

Email: ines.semendric@adelaide.edu.au

Online Resource 1: Search strategy

Search conducted on MEDLINE via PubMed, June 2021.

Search	Query	Records retrieved
#1	"child"[mh] OR "students"[mh:noexp] OR pediatric[tiab] OR paediatric[tiab] OR adolescen*[tiab] OR teen*[tiab] OR youth[tiab] OR child*[tiab] AND (english[Filter]) AND (allchild[Filter])	1,798,047
#2	"cancer survivors"[mh] OR "brain neoplasms/psychology"[mh] OR "brain neoplasms/rehabilitation"[mh] OR "leukemia/psychology"[mh] OR "leukemia/rehabilitation"[mh] OR childhood cancer survivors[tiab] OR brain tumor*[tiab] OR brain tumour*[tiab] OR cancer[tiab] OR pediatric oncology[tiab] OR paediatric oncology[tiab] OR childhood brain tumor survivor*[tiab] OR childhood brain tumour survivor*[tiab] OR neurological tumor*[tiab] OR neurological tumour*[tiab] AND (english[Filter]) AND (allchild[Filter])	88,437

**#3**	"socialization"[mh] OR "interpersonal relations"[mh:noexp] OR "social integration"[mh] OR "social interaction"[mh] OR "social skills"[mh] OR "peer group"[mh] OR "schools"[mh:noexp] OR "social adjustment"[mh] OR "adaptation, psychological"[mh] OR "cooperative behavior"[mh] OR "child development"[mh] OR "family relations/psychology"[mh] OR "social participation"[mh] OR "child behavior"[mh] OR "social support"[mh] OR "quality of life/psychology"[mh] OR "school health services"[mh:noexp] OR "school mental health services"[mh] OR "academic performance/psychology"[mh] OR Survivorship[tiab] OR "school re-entry"[tiab] OR socialisation[tiab] OR socialization[tiab] OR social rules[tiab] OR reintegration[tiab] OR social challenge*[tiab] OR school AND (english[Filter]) AND (allchild[Filter])	**820,948**
**#4**	"qualitative research"[mh] OR "interviews as topic"[mh] OR "surveys and questionnaires"[mh] OR "focus groups"[mh] OR "cross-sectional studies"[mh] OR qualitative[tiab] OR interview*[tiab] OR narrative*[tiab] OR questionnaire*[tiab] OR thematic analys*[tiab] AND (english[Filter]) AND (allchild[Filter])	**500,697**
**#5**	"peripheral nervous system diseases"[mesh] OR "middle aged"[mesh] OR "Randomized Controlled Trial" [Publication Type] OR "Review" [Publication Type] OR "Review Literature as Topic"[mesh] OR “Ambulatory Care”[mesh] OR “Grief*”[mesh] OR “Sarcoma”[mesh] OR “breast neoplasms”[mesh] OR systematic review OR survivorship clinic OR dental OR fatigue OR financial OR infertility OR fertility OR vaccination OR influenza OR hearing loss OR sexual function OR breast cancer AND (english[Filter]) AND (allchild[Filter])	**1,308,416**
**#6**	**#1 AND #2 AND #3 AND #4 NOT #5**	**1,402**

Search strategy (MEDLINE via Ovid)

Search conducted on MEDLINE via Ovid, June 2021

**Search**	**Query**	**Records retrieved**
**#1**	exp child/ or students.sh. or pediatric.ti,ab. or paediatric.ti,ab. or adolescen.ti,ab. or teen*.ti,ab. or youth.ti,ab. or child*.ti,ab.	**2,628,951**
**#2**	exp cancer survivors/ or exp brain neoplasms/ or exp brain neoplasms/ or exp leukemia/ or exp leukemia/ or childhood cancer survivors.ti,ab. or brain tumor*.ti,ab. or brain tumour.ti,ab. or cancer.ti,ab. or pediatric oncology.ti,ab. or paediatric oncology.ti,ab. or childhood brain tumor survivor*.ti,ab. or childhood brain tumour survivor*.ti,ab. or neurological tumor*.ti,ab. or neurological tumour*.ti,ab.	**2,149,681**
**#3**	exp socialization/ or interpersonal relations.sh. or exp social integration/ or exp social interaction/ or exp social skills/ or exp peer group/ or schools.sh. or exp social adjustment/ or exp adaptation, psychological/ or exp cooperative behavior/ or exp child development/ or exp family relations/ or exp social participation/ or exp child behavior/ or exp social support/ or exp quality of life/ or school health services.sh. or exp school mental health services/ or exp academic performance/ or Survivorship.ti,ab. or school re-entry.ti,ab. or socialisation.ti,ab. or socialization.ti,ab. or social rules.ti,ab. or reintegration.ti,ab. or social challenge*.ti,ab. or school enrollment.ti,ab. or social adjustment*.ti,ab. or support at school.ti,ab. or school participation.ti,ab. or school life.ti,ab. or leisure time.ti,ab. or school readjustment.ti,ab. or neurocognitive.ti,ab. or acquiring knowledge.ti,ab. or applying	**1,078,395**

	knowledge.ti,ab. or executive functioning.ti,ab. or social life.ti,ab. or daily life.ti,ab. or psychological wellbeing.ti,ab. or psychological well being.ti,ab. or social functioning.ti,ab. or self care.ti,ab. or social functioning.ti,ab. or return to school.ti,ab. or returning to school.ti,ab. or academic.ti,ab. or school.ti,ab. or learning difficulties.ti,ab.	
**#4**	((exp qualitative research/ or exp interviews as topic/ or exp surveys/) and questionnaires.mp.) or exp focus groups/ or exp cross-sectional studies/ or qualitative.ti,ab. or interview*.ti,ab. or narrative*.ti,ab. or questionnaire*.ti,ab. or thematic analys*.ti,ab. [mp=title, abstract, original title, name of substance word, subject heading word, floating sub-heading word, keyword heading word, organism supplementary concept word, protocol supplementary concept word, rare disease supplementary concept word, unique identifier, synonyms]	**1,495,340**
**#5**	exp peripheral nervous system diseases/ or exp middle aged/ or exp Review Literature as Topic/ or exp Ambulatory Care/ or exp Sarcoma/ or exp breast neoplasms/ or systematic review.ti,ab. or Survivorship clinic.ti,ab. or dental.ti,ab. or fatigue.ti,ab. or financial.ti,ab. or infertility.ti,ab. or fertility.ti,ab. or vaccination.ti,ab. or influenza.ti,ab. or hearing loss.ti,ab. or sexual function.ti,ab. or breast cancer.ti,ab	**5,808,620**
**#6**	(1 and 2 and 3 and 4) not 5	**2,138**

Search strategy (Embase)

Search conducted on Embase, June 2021

Search	Query	Records retrieved
#1	"child"/exp OR pediatric:ti,ab OR paediatric:ti,ab OR adolescen*:ti,ab OR teen*:ti,ab OR youth:ti,ab OR child*:ti,ab	3,833,039
#2	"cancer survivor"/exp OR "childhood cancer survivors":ti,ab OR cancer:ti,ab OR "pediatric oncology":ti,ab OR "paediatric oncology":ti,ab	2,580,517
#3	"socialization"/exp OR "school"/de OR "child development"/exp OR "family relation"/exp OR "child behavior"/exp OR "social support"/exp OR "quality of life"/exp OR "school health service"/de OR "academic achievement"/exp OR "school re-entry":ti,ab OR socialisation:ti,ab OR socialization:ti,ab OR reintegration:ti,ab OR "social adjustment*":ti,ab OR "support at school":ti,ab OR "school life":ti,ab OR "leisure time":ti,ab OR "school readjustment":ti,ab OR neurocognitive:ti,ab OR "executive functioning":ti,ab OR "social life":ti,ab OR "daily life":ti,ab OR "return to school":ti,ab OR "returning to school":ti,ab OR academic:ti,ab OR school:ti,ab OR "learning difficulties":ti,ab	1,404,008
#4	"qualitative research"/exp OR "interview "/exp OR "questionnaire"/exp OR "cross-sectional study"/exp OR surveys:ti,ab OR qualitative:ti,ab OR "focus group":ti,ab OR interview*:ti,ab OR narrative*:ti,ab OR questionnaire*:ti,ab OR "thematic analys*":ti,ab	2,032,597

**#5**	"chemotherapy-induced peripheral neuropathy"/exp OR "middle aged"/exp OR "randomized controlled trial"/exp OR "Review"/it OR “Ambulatory Care”/exp OR “Grief”/exp OR “Sarcoma”/exp OR “breast tumor”/exp OR systematic review:ti OR "Survivorship clinic":ti,ab OR dental:ti,ab OR fatigue:ti,ab OR financial:ti,ab OR infertility:ti,ab OR fertility:ti,ab OR vaccination:ti,ab OR influenza:ti,ab OR "hearing loss":ti,ab OR "sexual function":ti,ab OR "breast cancer":ti,ab OR “end of life”:ti,ab OR “adult survivors”:ti,ab OR “genetic”:ti,ab OR “spirituality”:ti,ab	**2,921,603**
**#6**	**#1 AND #2 AND #3 AND #4 NOT #5 AND** ([adolescent]/lim OR [child]/lim OR [preschool]/lim OR [school]/lim)	**2,705**

Search strategy (PsycINFO)

Search conducted on PsycINFO, June 2021

Search	Query	Records retrieved
**#1**	students.sh. or pediatric.ti,ab. or paediatric.ti,ab. or adolescen.ti,ab. or teen*.ti,ab. or youth.ti,ab. or child*.ti,ab	**8,099,57**
**#2**	exp brain neoplasms/ or exp brain neoplasms/ or exp leukemia/ or exp leukemia/ or childhood cancer survivors.ti,ab. or brain tumor*.ti,ab. or brain tumour.ti,ab. or cancer.ti,ab. or pediatric oncology.ti,ab. or paediatric oncology.ti,ab. or childhood brain tumor survivor*.ti,ab. or childhood brain tumour survivor*.ti,ab. or neurological tumor*.ti,ab. or neurological tumour*.ti,ab	**66,966**
**#3**	exp socialization/ or interpersonal relations.sh. or exp social integration/ or exp social interaction/ or exp social skills/ or schools.sh. or exp social adjustment/ or exp family relations/ or exp child behavior/ or exp social support/ or exp quality of life/ or school health services.sh. or Survivorship.ti,ab. or school re-entry.ti,ab. or socialisation.ti,ab. or socialization.ti,ab. or social rules.ti,ab. or reintegration.ti,ab. or social challenge*.ti,ab. or school enrollment.ti,ab. or social adjustment*.ti,ab. or support at school.ti,ab. or school participation.ti,ab. or school life.ti,ab. or leisure time.ti,ab. or school readjustment.ti,ab. or neurocognitive.ti,ab. or acquiring knowledge.ti,ab. or applying knowledge.ti,ab. or executive functioning.ti,ab. or social life.ti,ab. or daily life.ti,ab. or psychological wellbeing.ti,ab. or psychological well being.ti,ab. or social functioning.ti,ab. or self care.ti,ab. or social	**1,170,070**

	functioning.ti,ab. or return to school.ti,ab. or returning to school.ti,ab. or academic.ti,ab. or school.ti,ab. or learning difficulties.ti,ab	
**#4**	((exp qualitative research/ or exp interviews/ or exp surveys/) and questionnaires.mp.) or exp focus group/ or cross-sectional studies.ti,ab. or qualitative.ti,ab. or interview*.ti,ab. or narrative*.ti,ab. or questionnaire*.ti,ab. or thematic analys*.ti,ab. [mp=title, abstract, heading word, table of contents, key concepts, original title, tests & measures, mesh]	**7,213,22**
**#5**	exp Ambulatory Care/ or exp Sarcoma/ or exp breast neoplasms/ or systematic review.ti,ab. or Survivorship clinic.ti,ab. or dental.ti,ab. or fatigue.ti,ab. or financial.ti,ab. or infertility.ti,ab. or fertility.ti,ab. or vaccination.ti,ab. or influenza.ti,ab. or hearing loss.ti,ab. or sexual function.ti,ab. or breast cancer.ti,ab	**1,927,25**
**#6**	**(1 and 2 and 3 and 4) not 5**	**145**

Search strategy (CINAHL)

Search conducted on CINAHL, June 2021

Search	Query	Records retrieved
**#1**	MH "child+" OR MH "students" OR MH "Students, Middle School" OR TI,AB "pediatric" OR TI,AB "paediatric" OR TI,AB "adolescen*" OR TI,AB "teen*" OR TI,AB "youth" OR TI,AB "child*"	**701,210**
**#2**	MH "cancer survivors+" OR MH "brain neoplasms+" OR MH "children neoplasms+" OR MH "leukemia+" OR TI,AB "childhood cancer survivors" OR TI,AB "Brain tumor*" OR TI,AB "brain tumour*" OR TI,AB "cancer" OR TI,AB "pediatric oncology" OR TI,AB "paediatric oncology" OR TI,AB "childhood brain tumor survivor*" OR TI,AB "childhood brain tumour survivor*" OR TI,AB "neurological tumor*" OR TI,AB "neurological tumour*"	**48,707**
**#3**	MH "socialization+" OR MH "interpersonal relations+" OR MH "Psychology, Social+" OR MH "Schools" OR MH "social adjustment+" OR MH "adaptation, psychological+" OR MH "cooperative behavior+" OR MH "family relations+ " OR MH "social participation+" OR MH "child behavior+" OR MH "support, psychosocial+" OR MH "quality of life+" OR MH "school health services" OR MH "school mental health services" OR MH "academic performance+" OR TI,AB "Survivorship" OR TI,AB "school re-entry" OR TI,AB "socialisation" OR TI,AB "socialization" OR TI,AB "social rules" OR TI,AB "reintegration" OR TI,AB "social challenge*" OR TI,AB "school	**1,393,526**

	enrolment" OR TI,AB "social adjustment*" OR TI,AB "support at school" OR TI,AB "school participation" OR TI,AB "school life" OR TI,AB "leisure time" OR TI,AB "school readjustment" OR TI,AB "neurocognitive" OR TI,AB "acquiring knowledge" OR TI,AB "applying knowledge" OR TI,AB "executive function*" OR TI,AB "social life" OR TI,AB "daily life" OR TI,AB "psychological wellbeing" OR TI,AB "psychological well being" OR TI,AB "social functioning" OR TI,AB "self care" OR TI,AB "social functioning" OR TI,AB "return to school" OR TI,AB "returning to school" OR TI,AB "academic" OR TI,AB "school" OR TI,AB "learning difficulties" OR TI,AB "child development"	
**#4**	MH "qualitative studies+" OR MH "interviews+" OR MH "semi-structured interview+" OR MH "surveys+" OR MH "focus groups+" OR MH "cross sectional studies+" OR TI,AB "qualitative" OR TI,AB "interview*" OR TI,AB "narrative*" OR TI,AB "questionnaire*" OR TI,AB "thematic analys*"	**664,359**
**#5**	MH "peripheral nervous system diseases+" OR MH "middle age+" OR MH "Randomized Controlled Trials+" OR MH "Systematic Review+" OR MH "Literature Review+" OR MH "Ambulatory Care+" OR MH "Grief+" OR MH "Sarcoma+" OR MH "breast neoplasms+" OR TI,AB "systematic review" OR TI,AB "Survivorship clinic" OR TI,AB "dental" OR TI,AB "fatigue" OR TI,AB "financial" OR TI,AB "infertility" OR TI,AB "fertility" OR TI,AB "vaccination" OR TI,AB	**1,316,104**

	"influenza" OR TI,AB "hearing loss" OR TI,AB "sexual function" OR TI,AB "breast cancer"	
**#6**	**#1 AND #2 AND #3 AND #4 NOT #5**	**327**

Search strategy (ProQuest Dissertations & Theses)

Search conducted on ProQuest Dissertations & Theses: Global, June 2021

Search	Query	Records retrieved
**#1**	Noft(Child OR students OR pediatric OR paediatric OR adolescent OR teen OR youth OR child)	**586,011**
**#2**	Noft((cancer survivors) OR (brain neoplasms) OR (brain neoplasms) OR leukemia OR (childhood cancer survivors) OR (brain tumor) OR (brain tumour) OR Cancer OR (pediatric oncology) OR (paediatric oncology) OR (childhood brain tumor survivor) OR (childhood brain tumour survivor) OR (neurological tumor) OR (neurological tumour))	**91,343**
**#3**	Noft(socialization OR (interpersonal relations) OR (social integration) OR (social interaction) OR (social skills) OR (peer group) OR schools OR (social adjustment) OR (adaptation, psychological) OR (cooperative behavior) OR (child development) OR (family relations) OR (social participation) OR (child behavior) OR (social support) OR (quality of life) OR (school health services) OR (school mental health services) OR (academic performance) OR Survivorship OR (school re-entry) OR socialisation OR socialization OR (social rules) OR reintegration OR (social challenge) OR (school enrolment) OR (social adjustment) OR (support at school) OR (school participation) OR (school life) OR	**1,203,828**

	(leisure time) OR (school readjustment) OR neurocognitive OR (acquiring knowledge) OR (applying knowledge) OR (executive functioning) OR (social life) OR (daily life) OR (psychological wellbeing) OR (psychological well being) OR (social functioning) OR (self care) OR (social functioning) OR (return to school) OR (returning to school) OR academic OR school OR (learning difficulties))	
**#4**	Noft((qualitative research) OR (interviews as topic) OR (surveys and questionnaires) OR (focus groups) OR (cross-sectional studies) OR qualitative OR interview OR narrative OR questionnaire OR (thematic analysis))	**645,949**
**#5**	(peripheral nervous system diseases) OR (middle aged) OR (Randomized Controlled Trial) OR Review OR (Review Literature as Topic) OR (Ambulatory Care) OR Grief OR Sarcoma OR (breast neoplasms) OR (systematic review) OR (Survivorship clinic) OR dental OR fatigue OR financial OR infertility OR fertility OR vaccination OR influenza OR (hearing loss) OR (sexual function) OR (breast cancer)	**2,406,599**
**#6**	**#1 AND #2 AND #3 AND #4 NOT #5**	**170**

Updated Search strategy

Search conducted on MEDLINE via PubMed, 28th June 2022

Search	Query	Records retrieved
**#1**	"child"[mh] OR "students"[mh:noexp] OR pediatric[tiab] OR paediatric[tiab] OR adolescen*[tiab] OR teen*[tiab] OR youth[tiab] OR child*[tiab] AND (english[Filter]) AND (allchild[Filter])	**67,293**
**#2**	"cancer survivors"[mh] OR "brain neoplasms/psychology"[mh] OR "brain neoplasms/rehabilitation"[mh] OR "leukemia/psychology"[mh] OR "leukemia/rehabilitation"[mh] OR childhood cancer survivors[tiab] OR brain tumor*[tiab] OR brain tumour*[tiab] OR cancer[tiab] OR pediatric oncology[tiab] OR paediatric oncology[tiab] OR childhood brain tumor survivor*[tiab] OR childhood brain tumour survivor*[tiab] OR neurological tumor*[tiab] OR neurological tumour*[tiab] AND (english[Filter]) AND (allchild[Filter])	**4,019**
**#3**	"socialization"[mh] OR "interpersonal relations"[mh:noexp] OR "social integration"[mh] OR "social interaction"[mh] OR "social skills"[mh] OR "peer group"[mh] OR "schools"[mh:noexp] OR "social adjustment"[mh] OR "adaptation, psychological"[mh] OR "cooperative behavior"[mh] OR "child development"[mh] OR "family relations/psychology"[mh] OR "social participation"[mh] OR "child behavior"[mh] OR "social support"[mh] OR "quality of life/psychology"[mh] OR "school health services"[mh:noexp] OR "school mental health services"[mh] OR "academic performance/psychology"[mh] OR Survivorship[tiab] OR "school re-entry"[tiab] OR socialisation[tiab] OR socialization[tiab] OR	**46,222**

	social rules[tiab] OR reintegration[tiab] OR social challenge*[tiab] OR school AND (english[Filter]) AND (allchild[Filter])	
**#4**	"qualitative research"[mh] OR "interviews as topic"[mh] OR "surveys and questionnaires"[mh] OR "focus groups"[mh] OR "cross-sectional studies"[mh] OR qualitative[tiab] OR interview*[tiab] OR narrative*[tiab] OR questionnaire*[tiab] OR thematic analys*[tiab] AND (english[Filter]) AND (allchild[Filter])	**24,092**
**#5**	"peripheral nervous system diseases"[mesh] OR "middle aged"[mesh] OR "Randomized Controlled Trial" [Publication Type] OR "Review" [Publication Type] OR "Review Literature as Topic"[mesh] OR “Ambulatory Care”[mesh] OR “Grief*”[mesh] OR “Sarcoma”[mesh] OR “breast neoplasms”[mesh] OR systematic review OR survivorship clinic OR dental OR fatigue OR financial OR infertility OR fertility OR vaccination OR influenza OR hearing loss OR sexual function OR breast cancer AND (english[Filter]) AND (allchild[Filter])	**38,380**
**#6**	**#1 AND #2 AND #3 AND #4 NOT #5**	**209**
Date range: 28th June 2021-today		

Search strategy (MEDLINE via Ovid)

Search conducted on MEDLINE via Ovid, 28th June 2022

Search	Query	Records retrieved
#1	exp child/ or students.sh. or pediatric.ti,ab. or paediatric.ti,ab. or adolescen.ti,ab. or teen*.ti,ab. or youth.ti,ab. or child*.ti,ab.	**2,753,408**
#2	exp cancer survivors/ or exp brain neoplasms/ or exp brain neoplasms/ or exp leukemia/ or exp leukemia/ or childhood cancer survivors.ti,ab. or brain tumor*.ti,ab. or brain tumour.ti,ab. or cancer.ti,ab. or pediatric oncology.ti,ab. or paediatric oncology.ti,ab. or childhood brain tumor survivor*.ti,ab. or childhood brain tumour survivor*.ti,ab. or neurological tumor*.ti,ab. or neurological tumour*.ti,ab.	**2,303,422**
#3	exp socialization/ or interpersonal relations.sh. or exp social integration/ or exp social interaction/ or exp social skills/ or exp peer group/ or schools.sh. or exp social adjustment/ or exp adaptation, psychological/ or exp cooperative behavior/ or exp child development/ or exp family relations/ or exp social participation/ or exp child behavior/ or exp social support/ or exp quality of life/ or school health services.sh. or exp school mental health services/ or exp academic performance/ or Survivorship.ti,ab. or school re-entry.ti,ab. or socialisation.ti,ab. or socialization.ti,ab. or social rules.ti,ab. or reintegration.ti,ab. or social challenge*.ti,ab. or school enrollment.ti,ab. or social adjustment*.ti,ab. or support at school.ti,ab. or school participation.ti,ab. or school life.ti,ab. or leisure time.ti,ab. or school readjustment.ti,ab. or neurocognitive.ti,ab. or acquiring knowledge.ti,ab. or applying knowledge.ti,ab. or executive functioning.ti,ab. or social life.ti,ab. or	**1,165,875**

	daily life.ti,ab. or psychological wellbeing.ti,ab. or psychological well being.ti,ab. or social functioning.ti,ab. or self care.ti,ab. or social functioning.ti,ab. or return to school.ti,ab. or returning to school.ti,ab. or academic.ti,ab. or school.ti,ab. or learning difficulties.ti,ab.	
**#4**	((exp qualitative research/ or exp interviews as topic/ or exp surveys/) and questionnaires.mp.) or exp focus groups/ or exp cross-sectional studies/ or qualitative.ti,ab. or interview*.ti,ab. or narrative*.ti,ab. or questionnaire*.ti,ab. or thematic analys*.ti,ab. [mp=title, abstract, original title, name of substance word, subject heading word, floating sub-heading word, keyword heading word, organism supplementary concept word, protocol supplementary concept word, rare disease supplementary concept word, unique identifier, synonyms]	**1,652,360**
**#5**	exp peripheral nervous system diseases/ or exp middle aged/ or exp Review Literature as Topic/ or exp Ambulatory Care/ or exp Sarcoma/ or exp breast neoplasms/ or systematic review.ti,ab. or Survivorship clinic.ti,ab. or dental.ti,ab. or fatigue.ti,ab. or financial.ti,ab. or infertility.ti,ab. or fertility.ti,ab. or vaccination.ti,ab. or influenza.ti,ab. or hearing loss.ti,ab. or sexual function.ti,ab. or breast cancer.ti,ab	**6,111,949**
**#6**	(1 and 2 and 3 and 4) not 5 limit to dt=20210401-20220328<br>*Date range: 28th June 2021-today*	**175**

Search strategy (Embase)

Search conducted on Embase via Ovid, 28th June 2022

Search strategy had to be amended as Embase has moved from Elsevier to Ovid.

Search	Query	Records retrieved
#1	exp child/ OR pediatric:ti,ab OR paediatric:ti,ab OR adolescen*:ti,ab OR teen*:ti,ab OR youth:ti,ab OR child*:ti,ab	2,860,624
#2	exp "cancer survivor"/ OR "childhood cancer survivors:ti,ab" OR cancer.mp. OR "pediatric oncology:ti,ab" OR "paediatric oncology:ti,ab"	4,038,596
#3	exp "socialization"/ OR "school"/de OR exp "child development"/ OR exp "family relation"/ OR exp "child behavior"/ OR exp "social support"/ OR exp "quality of life"/ OR "school health service"/de OR exp "academic achievement"/ OR "school re-entry":ti,ab OR socialisation:ti,ab OR socialization:ti,ab OR reintegration:ti,ab OR "social adjustment*":ti,ab OR "support at school":ti,ab OR "school life":ti,ab OR "leisure time":ti,ab OR "school readjustment":ti,ab OR neurocognitive:ti,ab OR "executive functioning":ti,ab OR "social life":ti,ab OR "daily life":ti,ab OR "return to school":ti,ab OR "returning to school":ti,ab OR academic:ti,ab OR school:ti,ab OR "learning difficulties":ti,ab	885,943
#4	exp "qualitative research"/ OR exp "interview "/ OR exp "questionnaire"/ OR exp "cross-sectional study"/ OR surveys:ti,ab OR qualitative:ti,ab OR "focus group":ti,ab OR interview*:ti,ab OR narrative*:ti,ab OR questionnaire*:ti,ab OR "thematic analys*":ti,ab	1,497,439

**#5**	exp "chemotherapy-induced peripheral neuropathy"/ OR exp "middle aged"/ OR exp randomized controlled trial/ OR exp "review"/ OR exp ambulatory care/ OR exp grief/ OR exp sarcoma/ OR exp breast tumor/ OR systematic review:ti OR survivorship clinic:ti,ab OR dental:ti,ab OR fatigue:ti,ab OR financial:ti,ab OR infertility:ti,ab OR fertility:ti,ab OR vaccination:ti,ab OR influenza:ti,ab OR hearing loss:ti,ab OR sexual function:ti,ab OR breast cancer:ti,ab OR end of life:ti,ab OR adult survivors:ti,ab OR genetic:ti,ab OR spirituality:ti,ab	**5,931,113**
**#6**	(1 and 2 and 3 and 4) not 5 limit to dd=20210401-20220328<br>*Date range: 28th June 2021-today*	**68**

Search strategy (PsycINFO)

Search conducted on PsycINFO, 28th June 2022

Search	Query	Records retrieved
**#1**	students.sh. or pediatric.ti,ab. or paediatric.ti,ab. or adolescen.ti,ab. or teen*.ti,ab. or youth.ti,ab. or child*.ti,ab	**843,316**
**#2**	exp brain neoplasms/ or exp brain neoplasms/ or exp leukemia/ or exp leukemia/ or childhood cancer survivors.ti,ab. or brain tumor*.ti,ab. or brain tumour.ti,ab. or cancer.ti,ab. or pediatric oncology.ti,ab. or paediatric oncology.ti,ab. or childhood brain tumor survivor*.ti,ab. or childhood brain tumour survivor*.ti,ab. or neurological tumor*.ti,ab. or neurological tumour*.ti,ab	**70,376**
**#3**	exp socialization/ or interpersonal relations.sh. or exp social integration/ or exp social interaction/ or exp social skills/ or schools.sh. or exp social adjustment/ or exp family relations/ or exp child behavior/ or exp social support/ or exp quality of life/ or school health services.sh. or Survivorship.ti,ab. or school re-entry.ti,ab. or socialisation.ti,ab. or socialization.ti,ab. or social rules.ti,ab. or reintegration.ti,ab. or social challenge*.ti,ab. or school enrollment.ti,ab. or social adjustment*.ti,ab. or support at school.ti,ab. or school participation.ti,ab. or school life.ti,ab. or leisure time.ti,ab. or school readjustment.ti,ab. or neurocognitive.ti,ab. or acquiring knowledge.ti,ab. or applying knowledge.ti,ab. or executive functioning.ti,ab. or social life.ti,ab. or daily life.ti,ab. or psychological wellbeing.ti,ab. or psychological well being.ti,ab. or social functioning.ti,ab. or self care.ti,ab. or social	**1,228,259**

	functioning.ti,ab. or return to school.ti,ab. or returning to school.ti,ab. or academic.ti,ab. or school.ti,ab. or learning difficulties.ti,ab	
**#4**	((exp qualitative research/ or exp interviews/ or exp surveys/) and questionnaires.mp.) or exp focus group/ or cross-sectional studies.ti,ab. or qualitative.ti,ab. or interview*.ti,ab. or narrative*.ti,ab. or questionnaire*.ti,ab. or thematic analys*.ti,ab. [mp=title, abstract, heading word, table of contents, key concepts, original title, tests & measures, mesh]	**763,894**
**#5**	exp Ambulatory Care/ or exp Sarcoma/ or exp breast neoplasms/ or systematic review.ti,ab. or Survivorship clinic.ti,ab. or dental.ti,ab. or fatigue.ti,ab. or financial.ti,ab. or infertility.ti,ab. or fertility.ti,ab. or vaccination.ti,ab. or influenza.ti,ab. or hearing loss.ti,ab. or sexual function.ti,ab. or breast cancer.ti,ab	**206,337**
**#6**	(1 and 2 and 3 and 4) not 5 limit to up=20210401-20220328<br>*Date range: 28th June 2021-today*	**21**

Search strategy (CINAHL)

Search conducted on CINAHL, 28th June 2022

Search	Query	Records retrieved
#1	MH "child+" OR MH "students" OR MH "Students, Middle School" OR TI,AB "pediatric" OR TI,AB "paediatric" OR TI,AB "adolescen*" OR TI,AB "teen*" OR TI,AB "youth" OR TI,AB "child*"	737,178
#2	MH "cancer survivors+" OR MH "brain neoplasms+" OR MH "children neoplasms+" OR MH "leukemia+" OR TI,AB "childhood cancer survivors" OR TI,AB "Brain tumor*" OR TI,AB "brain tumour*" OR TI,AB "cancer" OR TI,AB "pediatric oncology" OR TI,AB "paediatric oncology" OR TI,AB "childhood brain tumor survivor*" OR TI,AB "childhood brain tumour survivor*" OR TI,AB "neurological tumor*" OR TI,AB "neurological tumour*"	52,236
#3	MH "socialization+" OR MH "interpersonal relations+" OR MH "Psychology, Social+" OR MH "Schools" OR MH "social adjustment+" OR MH "adaptation, psychological+" OR MH "cooperative behavior+" OR MH "family relations+ " OR MH "social participation+" OR MH "child behavior+" OR MH "support, psychosocial+" OR MH "quality of life+" OR MH "school health services" OR MH "school mental health services" OR MH "academic performance+" OR TI,AB "Survivorship" OR TI,AB "school re-entry" OR TI,AB "socialisation" OR TI,AB "socialization" OR TI,AB "social rules" OR TI,AB "reintegration" OR TI,AB "social challenge*" OR TI,AB "school enrolment" OR TI,AB "social adjustment*" OR TI,AB "support at school" OR TI,AB "school participation" OR TI,AB "school life" OR	1,477,450

	TI,AB “leisure time” OR TI,AB “school readjustment” OR TI,AB “neurocognitive” OR TI,AB “acquiring knowledge” OR TI,AB “applying knowledge” OR TI,AB “executive function*” OR TI,AB “social life” OR TI,AB “daily life” OR TI,AB “psychological wellbeing” OR TI,AB “psychological well being” OR TI,AB “social functioning” OR TI,AB “self care” OR TI,AB “social functioning” OR TI,AB “return to school” OR TI,AB “returning to school” OR TI,AB “academic” OR TI,AB “school” OR TI,AB “learning difficulties” OR TI,AB “child development”	
**#4**	MH "qualitative studies+" OR MH "interviews+" OR MH “semi-structured interview+” OR MH "surveys+" OR MH "focus groups+" OR MH "cross sectional studies+" OR TI,AB “qualitative” OR TI,AB “interview*” OR TI,AB “narrative*” OR TI,AB “questionnaire*” OR TI,AB “thematic analys*”	**711,769**
**#5**	MH "peripheral nervous system diseases+" OR MH "middle age+" OR MH "Randomized Controlled Trials+" OR MH "Systematic Review+" OR MH "Literature Review+" OR MH “Ambulatory Care+” OR MH “Grief+” OR MH “Sarcoma+” OR MH “breast neoplasms+” OR TI,AB “systematic review” OR TI,AB “Survivorship clinic” OR TI,AB “dental” OR TI,AB “fatigue” OR TI,AB “financial” OR TI,AB “infertility” OR TI,AB “fertility” OR TI,AB “vaccination” OR TI,AB “influenza” OR TI,AB “hearing loss” OR TI,AB “sexual function” OR TI,AB “breast cancer”	**1,402,605**
**#6**	(S1 AND S2 AND S3 AND S4) NOT S5<br>Limiters - Published Date: 20210601-20220628	**21**

Search strategy (ProQuest Dissertations & Theses)

Search conducted on ProQuest Dissertations & Theses: Global, 28th June 2022

Search	Query	Records retrieved
**#1**	Noft(Child OR students OR pediatric OR paediatric OR adolescent OR teen OR youth OR child)	631,187
**#2**	Noft((cancer survivors) OR (brain neoplasms) OR (brain neoplasms) OR leukemia OR (childhood cancer survivors) OR (brain tumor) OR (brain tumour) OR Cancer OR (pediatric oncology) OR (paediatric oncology) OR (childhood brain tumor survivor) OR (childhood brain tumour survivor) OR (neurological tumor) OR (neurological tumour))	**101,267**
**#3**	Noft(socialization OR (interpersonal relations) OR (social integration) OR (social interaction) OR (social skills) OR (peer group) OR schools OR (social adjustment) OR (adaptation, psychological) OR (cooperative behavior) OR (child development) OR (family relations) OR (social participation) OR (child behavior) OR (social support) OR (quality of life) OR (school health services) OR (school mental health services) OR (academic performance) OR Survivorship OR (school re-entry) OR socialisation OR socialization OR (social rules) OR reintegration OR (social challenge) OR (school enrolment) OR (social adjustment) OR (support at school) OR (school participation) OR (school life) OR	**1,281,723**

	(leisure time) OR (school readjustment) OR neurocognitive OR (acquiring knowledge) OR (applying knowledge) OR (executive functioning) OR (social life) OR (daily life) OR (psychological wellbeing) OR (psychological well being) OR (social functioning) OR (self care) OR (social functioning) OR (return to school) OR (returning to school) OR academic OR school OR (learning difficulties))	
**#4**	Noft((qualitative research) OR (interviews as topic) OR (surveys and questionnaires) OR (focus groups) OR (cross-sectional studies) OR qualitative OR interview OR narrative OR questionnaire OR (thematic analysis))	**703,240**
**#5**	(peripheral nervous system diseases) OR (middle aged) OR (Randomized Controlled Trial) OR Review OR (Review Literature as Topic) OR (Ambulatory Care) OR Grief OR Sarcoma OR (breast neoplasms) OR (systematic review) OR (Survivorship clinic) OR dental OR fatigue OR financial OR infertility OR fertility OR vaccination OR influenza OR (hearing loss) OR (sexual function) OR (breast cancer)	**2,613,627**
**#6**	**#1 AND #2 AND #3 AND #4 NOT #5**	**6**
Additional limits – Date: From 28 June 2021 to today		
