# Supplementary material for: “Chemobrain” in childhood cancer survivors—the impact on social, academic, and daily living skills: a qualitative systematic review
Source: Support Care Cancer. 2023 Aug 22;31(9):532. doi: 10.1007/s00520-023-07985-z (PMC10444646; doi:10.1007/s00520-023-07985-z)
Supplement: Supplementary file 2 — Supplementary file2 (PDF 65 KB) [file 520_2023_7985_MOESM2_ESM.pdf]

“Chemobrain” in childhood cancer survivors – the impact on social, academic, and daily living skills: a qualitative systematic review

Ines Semendric[1*], Danielle Pollock[2], Olivia J Haller[1], Rebecca P George[1], Lyndsey E. Collins-Praino[1], Alexandra Whittaker[3]

1. School of Biomedicine, The University of Adelaide, Adelaide, South Australia

2. JBI, Faculty of Health and Medical Sciences, Adelaide, South Australia

3. School of Animal and Veterinary Sciences, The University of Adelaide, Roseworthy, South Australia

*Corresponding author: Ines Semendric

Email: ines.semendric@adelaide.edu.au

Online Resource 2: Data Extraction Instrument

JBI data extraction instrument

Study title	Author(s)	Methods for data collection and analysis	Country	Phenomena of interest	Setting/context/culture	Participant characteristics and sample size	Description of main results
