# Supplementary material for: “Chemobrain” in childhood cancer survivors—the impact on social, academic, and daily living skills: a qualitative systematic review
Source: Support Care Cancer. 2023 Aug 22;31(9):532. doi: 10.1007/s00520-023-07985-z (PMC10444646; doi:10.1007/s00520-023-07985-z)
Supplement: Supplementary file 3 — Supplementary file3 (PDF 125 KB) [file 520_2023_7985_MOESM3_ESM.pdf]

“Chemobrain” in childhood cancer survivors – the impact on social, academic, and daily living skills: a qualitative systematic review

Ines Semendric[1*], Danielle Pollock[2], Olivia J Haller[1], Rebecca P George[1], Lyndsey E. Collins-Praino[1], Alexandra Whittaker[3]

1. School of Biomedicine, The University of Adelaide, Adelaide, South Australia

2. JBI, Faculty of Health and Medical Sciences, Adelaide, South Australia

3. School of Animal and Veterinary Sciences, The University of Adelaide, Roseworthy, South Australia

*Corresponding author: Ines Semendric

Email: ines.semendric@adelaide.edu.au

Online Resource 3: Unsupported findings

Study: Choquette 2016	
Finding	Effect of illness and the impact these have not the child- cognitive problems (N)
In-text	We spoke to [the clinical psychologist] and he did an IQ thing and they said she didn’t need it [special education]. But I think that in itself is unfair as well, because she was so bright before she got poorly, why should she be kept back? Just because she’s bright it doesn’t mean she’s going to catch up and get back to the same level as everybody else of her age, does it, when she’s had so much time out.
Study: Lopez 2011	

Finding	Chemobrain as an effect of treatment (N)
In-text	Notably, one adolescent, who was diagnosed with AML, reported experiencing “chemo brain” after treatment completion. Chemo brain is a phenomenon believed to reflect deficits in memory, attention, and other cognitive functions (Staat & Segatore, 2005). This adolescent reported some changes in memory function that impacted his ability to perform certain academic tasks such as memorizing information for quizzes and tests. However, he explained that chemo brain did not significantly impact his overall academic functioning and performance. He indicated that his memory function has improved over time.
