# Supplementary material for: “Chemobrain” in childhood cancer survivors—the impact on social, academic, and daily living skills: a qualitative systematic review
Source: Support Care Cancer. 2023 Aug 22;31(9):532. doi: 10.1007/s00520-023-07985-z (PMC10444646; doi:10.1007/s00520-023-07985-z)
Supplement: Supplementary file 4 — Supplementary file4 (PDF 200 KB) [file 520_2023_7985_MOESM4_ESM.pdf]

“Chemobrain” in childhood cancer survivors – the impact on social, academic, and daily living skills: a qualitative systematic review

Ines Semendric[1*], Danielle Pollock[2], Olivia J Haller[1], Rebecca P George[1], Lyndsey E. Collins-Praino[1], Alexandra Whittaker[3]

1. School of Biomedicine, The University of Adelaide, Adelaide, South Australia

2. JBI, Faculty of Health and Medical Sciences, Adelaide, South Australia

3. School of Animal and Veterinary Sciences, The University of Adelaide, Roseworthy, South Australia

*Corresponding author: Ines Semendric

Email: ines.semendric@adelaide.edu.au

Online Resource 4: Characteristics of Included Studies

Study	Methods for data collection and analysis	Country	Phenomena of interest	Setting/context/culture	Participant characteristics and sample size	Description of main results
Chen et al. 2015	This study used a descriptive phenomenological design (Wojnar & Swanson, 2007) to construct meaning-based	Taiwan	Describe the lived experiences of Taiwanese mothers and	Taiwanese culture. Participants were recruited from a Medical Center in Taiwan. All interviews were	Inclusion criteria: (1) adolescents were 12 to 18 years old and had completed cancer treatment more than 1	The relationship between adolescents with cancer and their mothers was represented by the overarching metaphorical

	nursing knowledge about the experiences of mothers and their adolescents returning to school after completing cancer treatment in Taiwan. Adolescents with cancer and their mothers were purposively sampled from a medical center in Taiwan by asking their physicians to screen them for inclusion criteria.<br><br>Data were obtained in 60- to 90-minute interviews that followed a semi-structured interview guide using open-		their adolescents who had completed cancer treatment and returned to school.	conducted in participant's homes.	year previously, (2) mothers were the primary caregiver and birth mother of the adolescent with cancer, (3) both mothers and adolescents spoke Mandarin or Taiwanese, (4) both agreed to be tape-recorded during interviews, and (5) both lived in the same family.<br><br>Exclusion criteria were (1) adolescent	theme of a machine with gears turning separately and then meshing to move forward together. After the adolescents completed their cancer treatment, they, and their mothers both experienced different distresses related to their social roles. At some point, they both set the same goal to move forward simultaneously. The experiences of mothers and their adolescents after cancer treatment were represented by 3

	ended questions and non-directive interviewing skills. To allow participants to fully express their experience, the mothers and their adolescents were interviewed separately. To ensure the integrity of the data collected, interviews were recorded with subjects' consent and supplemented with written notes on facial expressions, tone of voice, and nonverbal physical responses during the process. Respecting				or mother was cognitively impaired, (2) adolescent's health status was unstable, such as cancer recurrence or at a terminal stage, and (3) either adolescent or mother in the same family refused to join the study.<br><br>Of the 14 mother-adolescent dyads who met the criteria, only 11 dyads agreed to participate. Data were	phenomenological themes: (a) spinning alone, side by side, (b) meshing without fiction, and (c) moving forward together. To move toward a normal and healthy life journey, the mothers and their adolescent worked together as a well-tuned machine. Cancer survivors primarily identified changes in body structure and function, discrimination by classmates, and poor academic performance as

	participants' preferences, all data were collected in their homes. In addition, medical chart reviews of adolescents with cancer were processed to record disease history, types of treatments, and recent status of follow-up. Each participant's interview material (tape and transcript) was coded as follows. The first adolescent participant was coded "A," the second adolescent participant as "B," and so on. The first adolescent's mother was				considered to reach saturation by the first 3 authors when no new information was found during con-current data collection and data analysis (Morse, 1994). Data were saturated after 8 pairs of adolescents and their mothers were separately interviewed between February and May 2010. The other 3 dyads who had	challenges during their experiences.

	coded “A’s M,” and the second adolescent’s mother as “B’s M,” and so on. Data were considered to reach saturation when no new information was found during concurrent data collection and data analysis (Morse, 1994).<br><br>Data was analysed by (1) reading each text to obtain a whole sense of the phenomena, (2) extracting significant statements described by participants that were relevant to the research questions, (3)				agreed to participate were told that their participation was no longer needed because of data saturation.	

	categorized significant statements to disclose their meanings no matter whether they were explicit or implicit, (4) conceptualized significant statements in groups and related them to themes generated from each participant regardless of whether the themes were contradictory or not, (5) integrated these themes into an exhaustive description that encompassed each text, (6) conceptualized the exhaustive description into a statement of the					

	phenomenon, (7) validate outcome of step 6 by the participants. On the dyadic level, similarities and differences between themes from adolescents’ and mothers’ narratives in the same situation were examined.					
Choquette, Rennick and Lee, 2016	Purposive sampling using interview. Semi-structured and composed of open-ended questions. Photos brought to interview to scaffold conversations- one ‘good’ thing about school, other ‘not so good’. Data	Canada	Describe how the return to school affects adolescents' beliefs about themselves, self-identity, and social	Return to school for adolescents after cancer treatment in Canada. Recruited from outpatient hematology/oncology clinic at a university-affiliated, quaternary,	12 participants met study eligibility criteria and agreed to participate. One participant was lost to attrition, resulting in a final sample size of 11.	Three main themes emerged to suggest that the return to school hallmarked a pivotal transition, signalling the end of cancer treatment, and a welcome return to a sense of well-being: (a) being on the

	from the photo discussion were embedded within the interview data. Interviews were transcribed verbatim, and all textual data (transcripts and observational field notes) were read in their entirety. Open coding included the actual words used by the adolescent, and the labelling of emotions and other observations described in the field notes. Data were examined line-by-line to identify the processes involved in		relationships after cancer treatment.	paediatric hospital. Interviews were organised during clinic visits or out of school hours.	Eligible participants (1) were 13 to 17 years of age; (2) had completed cancer treatment (chemotherapy with or without radiotherapy) between 6 months and 2 years prior; (3) had received medical clearance to return to school; (4) were fluent in French or English; and (5) were attending high school full time, in the	right track to recovery; (b) bridging two worlds; and (c) establishing a new life at school.

	returning to school. As data coding was ongoing, codes were checked and verified through direct questions during subsequent interviews to verify the researchers’ ongoing interpretation of the data. Axial coding was conducted, and mutually exclusive categories were identified to reflect the adolescents’ beliefs about themselves, their self-identity, and their social relationships.				appropriate grade for their age (T1 year).	

Suntup 1999	The parents of each child were asked to complete the Barkley Developmental History Form (Barkley, 1990) to obtain a record of the child's development and previous social or academic difficulties. The parents were also asked to complete the Child Behavior Checklist (CBCL) (Achenbach, 1991). Each parent individually completed a checklist so as to obtain his or her own perspective of the child's functioning. During the	USA	To assess the cognitive and psychosocial needs of children with Acute Lymphoblastic Leukemia (ALL), and to explore how these needs can best be addressed by means of an appropriate reintegration program.	Children from private elementary schools in California were referred to the study by the Oncology/Hematology team at the Children's Hospital of Orange County (CHOC). Testing was conducted at the Health Psychology department at CHOC. Observations were made in school, home visits, and testing as above.	4 participants met the eligibility criteria and agreed to participate. 1 participant was lost due to feeling overwhelmed by their medical treatment. Therefore, 3 participants were included. Eligible participants were (1) aged 6-12, (2) currently entering grades 1 to 7, (3) a diagnosis of Acute Lymphoblastic Leukemia, (4) being	Nonspecific attention problems were noted, particularly with regards to auditory attention. Planning and organizational difficulties, along with some memory problems were also evident. It was not possible to determine the role an inconsistent attention level may have had in creating noted cognitive difficulties. In addition, various ongoing environmental stressors may also have impacted the functioning of the

	maintenance phase of the CNS prophylaxis, two individual 2-hour testing sessions were scheduled with each child. During the intake session with the parents and first session with the child, all consent forms were signed (see Appendices A, B and C). When meeting with the child for the first testing session, rapport was established, and following a clinical interview, each child was administered the Kinetic Family Drawing				treated via CNS Prophylaxis, (5) had less than 2 years of maintenance therapy, and (6) had received home schooling during any time away from school.	participants. Further, school absence, body image concerns, and cultural factors may play a role in the reintegration of these children.

	(KFD) in order to assess his or her social and emotional functioning. A Sentence Completion task and/or some Human Figure Drawings was also included in this testing session. During the second session the children were administered a short form of the Wechsler Intelligence Test for Children—Third Edition (WISC-HI) (Wechsler, 1991) obtain a measure of each child's level of cognitive functioning. Therefore, the					

	second testing session also involved the administration of A Developmental Neuropsychological Assessment (NEPSY). School performance from records. Structured and informal observational of child in school classroom. Teacher interview and clinical interview with child.					
Vance, Eiser and Home 2004	Parents were interviewed, using a semi-structured format, in their own homes. Each interview lasted approximately 1–1.5 hours.	UK	Examine functioning among a homogenous group of young	Participants were recruited from Yorkshire Regional Center for Pediatric Haematology and Oncology.	Inclusion criteria were diagnosis (medullo-blastoma), language (English-speaking) and that	Two major themes emerged from the analysis. These were (i) the effects of the illness and their impact on the child, and (ii) dealing

	The interview schedule focused on themes representing the child’s functioning. These themes have been identified in previous literature reviews concerning children with cancer (Eiser, 1998). Throughout the interview, the questions were tailored to fit each parent’s account, i.e. they were not restricted to a pre-determined list of questions. This allowed parents to discuss issues important to them rather than fit the inter-viewer’s		CNS tumor survivors and long-term consequences of cancer and treatment.	Interviews took place at the participant’s homes.	survivors were well and in remission. Data were provided by the caregiver(s) responsible for the child. Eight transcripts (five mothers, one father, two mother–father pairs) were included in this study.	with the uncertainty of the illness and how this impacts on the child and parents.

	preconceptions of surviving this type of tumor. Parents were also asked to discuss any worries and concerns they had for their child and how they dealt with those concerns. Analysis involved familiarisation with the transcripts. Implicit within IPA, the analyst's interpretation is central, hence the use of a single rater. 'key words were jotted down to capture the essential qualities that were pertinent to the effects of the illness on the child					

	(e.g.balance, fine and gross motor coordination). The second stage involved re-reading the transcripts noting down more abstract terms, or super ordinate categories (e.g. balance and coordination problems would be subsumed within the category of physical problems). In following the IPA approach, these themes were not predicted in advance, and were subsequently considered in relation to the existing literature.					

Vanclooster et al. 2021	Multiple case study with longitudinal follow-up at three pre-determined time-points over a two-year period (when children had returned to school for at least 6-months, second interview one year later, third interview two years later). Semi-structured interviews (30-90 minutes, audio-taped and transcribed verbatim). Medical records and school documents were consulted.<br><br>Theoretical framework (International Classification	Belgium	What are the experiences of childhood brain tumor survivors (CBTS) and key figures in their environment regarding school life after returning, and can these experiences be described according to an existing classification system?	Children attending school in mainstream education. Children and parent were recruited from academic hospitals UZ Brussel and UZ Gent.	5 children met the study eligibility criteria as a case study (female n=2, male n=3, ages 7-10). Diagnosis included pilocytic astrocytoma, anaplastic ependymoma, medulloblastoma, and low-grade glioma. Treatment included surgery, radiotherapy, chemotherapy, or a combination.	**ICF component 1: body functions**<br><br>This includes mental functions, sensory functions and pain, voice and speech functions, neuromusculoskeletal and movement-related functions.<br><br>**ICF component 2: activities and participation**<br><br>This includes learning and applying knowledge, general tasks and demands, mobility, self-care,

	of Functioning, Disability and Health – Children and Youth framework) for data analysis. Through qualitative analysis each theme was assigned to the closest ICF (framework) code.				Eligible participants were (1) between 6 and 12 years old, (2) had to attend the same school in mainstream education as before their illness on a full-time basis, (3) had to be back at school for longer than 6 months to ensure adequate readjustment, (4) completed cancer treatment, and (5) had a good prognosis. Children who had	interpersonal interactions and relationships, and major life areas.<br><br>**ICF component 3: environmental factors**<br><br>This includes products and technology, support and relationships, and attitudes.<br><br>**ICF component 4: personal factors**<br><br>This includes services, systems and policies.<br><br>**Primary themes** reported by children and parents

					returned to school more than 3 years ago were excluded to prevent memory bias. Type of brain tumor or medical treatment was not an inclusion/exclusion criterion as they did not focus on diagnosis or disease-specific consequences.	included school life and participation, peer relationships and friendships, performance and difficulties, psychoscocial functioning and wellbeing, attitude and approach at school, and communication and collaboration.

Walker et al. 2019	Parents completed a demographic questionnaire that included age, race/ethnicity, marital status, and family income. Clinical characteristics (ie, diagnosis, date of diagnosis, date treatment was completed) were extracted from the electronic medical record by the first author. Once the forms were returned, the adolescent was officially enrolled in the study and was recontacted by a member of the study team to schedule the	USA	To describe the experience of adolescents in the first year after completing chemotherapy and/or radiation, including worries and perceived challenges they faced as they attempted to return to their	Adolescent survivors were recruited from a pediatric cancer program in a childrens hospital in the Pacific Northwest of the United States. Interviews were conducted over the phone.	Of the 67 eligible participants who were identified by medical record review and were mailed an opt-out recruitment letter, 34 participants (51%) enrolled, and 29 (43%) completed data collection. Study participants were eligible if they were (a) between the ages of 12 and 18 years when treatment was	Seven domains and 18 categories organized the adolescent’s experience with early post-treatment survivorship.<br><br>DOMAIN 1: TRYING TO FEEL NORMAL AGAIN<br><br>Five categories comprised trying to feel normal again: (1) getting back into the swing of things, (2) getting back into school, (3) regaining my strength, (4) getting better gradually, and (5) keeping myself healthy.

	telephone interview. Interviews ranged in length between 15 and 60 minutes, were digitally audio recorded, transcribed verbatim by a trained transcriptionist, and verified for 100% accuracy. Interviewers were specially trained to ask open-ended questions using a specified guide and to invite the adolescent to fully elaborate their responses. Qualitative data analysis was guided by a multi step process of		lives as adolescents.		completed, (b)had a primary diagnosis of cancer, (c) were in remission and had completed chemotherapy and/or radiation treatment within the last year, and d) were able to read, speak, and understand English. Three groups of adolescent survivors were excluded because their early survivorship	DOMAIN 2: IT'S ALL GOOD<br><br>Domain 2 included 3 categories: (1) feeling relieved to have treatment over, (2) doing fine, and (3) feeling prepared to move forward.<br><br>DOMAIN 3: STILL STRUGGLING<br><br>Domain 3 included 4 categories: (1) having adverse effects, (2) feeling down, (3) expecting me to

	inductive content analysis using methods adapted from grounded theory The coding process involved 5 steps, each maintaining the survivors' exact words: (1) All texts from the transcribed interviews were unitized. Each unit was a direct quotation of a complete idea that included both a noun and verb, whether implicit or explicit. (2) Each unit of data was then open coded using a code that started with a gerund (eg,feeling				experiences were less generalizable compared with other adolescent survivors: (a) those who were treated for brain tumors because their transitional care at our center may have included neuropsychiatry and/or cognitive rehabilitation, (b) those who received hematopoietic cell transplant because their	go back to normal, and (4) having chemo brain.<br><br>DOMAIN 4: MY RELATIONSHIPS<br><br>Adolescents described how their relationships with family and friends changed during treatment, some positively and others negatively. This domain consisted of 3 categories: (1) being stronger and closer, (2) having rocky friendships, and (3) having amazing doctors and nurses.

	confused, being happy). (3) The open codes were then organized into an initial set of categories with descriptions that clearly differentiated each category. (4) Constant comparative analysis was then used to verify the distinctions between the categories and verify the categories and verify the accuracy of fit between each unit of analysis in each category. (5) The refined set of categories was then grouped into higher-order domains that describe the				immunosuppressive regimen precluded return to school and other normative activities, and (c) those who had surgical resection only (eg, thyroid carcinoma) because they did not have an extended period of treatment.	DOMAIN 5: CHANGING ME<br><br>Three categories comprised this domain: (1) having a different perspective, (2) trying to understand my experience, and (3) wishing people already knew.<br><br>DOMAIN 6: WORRYING ABOUT RELAPSE<br><br>DOMAIN 7: CROSSING MY FINGERS<br><br>Remaining positive despite worry of relapse.

	experience of early post-treatment for adolescent cancer survivors.					
