# Supplementary material for: “Chemobrain” in childhood cancer survivors—the impact on social, academic, and daily living skills: a qualitative systematic review
Source: Support Care Cancer. 2023 Aug 22;31(9):532. doi: 10.1007/s00520-023-07985-z (PMC10444646; doi:10.1007/s00520-023-07985-z)
Supplement: Supplementary file 5 — Supplementary file5 (PDF 170 KB) [file 520_2023_7985_MOESM5_ESM.pdf]

**"Chemobrain" in childhood cancer survivors – the impact on social, academic, and daily living skills: a qualitative systematic review**

Ines Semendric[1*], Danielle Pollock[2], Olivia J Haller[1], Rebecca P George[1], Lyndsey E. Collins-Praino[1], Alexandra Whittaker[3]

1. School of Biomedicine, The University of Adelaide, Adelaide, South Australia

2. JBI, Faculty of Health and Medical Sciences, Adelaide, South Australia

3. School of Animal and Veterinary Sciences, The University of Adelaide, Roseworthy, South Australia

*Corresponding author: Ines Semendric

Email: ines.semendric@adelaide.edu.au

**Online Resource 5: JBI Checklist for Qualitative Research Template and Outcomes for Included Study**

## JBI CRITICAL APPRAISAL CHECKLIST FOR QUALITATIVE RESEARCH

Reviewer________________________________________ Date________________________________

Author_________________________________________ Year_________ Record Number_________

| | | Yes | No | Unclear | Not applicable |
|---|---|---|---|---|---|
| 1. | Is there congruity between the stated philosophical perspective and the research methodology? | □ | □ | □ | □ |
| 2. | Is there congruity between the research methodology and the research question or objectives? | □ | □ | □ | □ |
| 3. | Is there congruity between the research methodology and the methods used to collect data? | □ | □ | □ | □ |
| 4. | Is there congruity between the research methodology and the representation and analysis of data? | □ | □ | □ | □ |
| 5. | Is there congruity between the research methodology and the interpretation of results? | □ | □ | □ | □ |
| 6. | Is there a statement locating the researcher culturally or theoretically? | □ | □ | □ | □ |
| 7. | Is the influence of the researcher on the research, and vice- versa, addressed? | □ | □ | □ | □ |
| 8. | Are participants, and their voices, adequately represented? | □ | □ | □ | □ |
| 9. | Is the research ethical according to current criteria or, for recent studies, and is there evidence of ethical approval by an appropriate body? | □ | □ | □ | □ |

10. Do the conclusions drawn in the research report flow from the analysis, or interpretation, of the data? □ □ □ □

Overall appraisal: Include □ Exclude □ Seek further info □

Comments (Including reason for exclusion)

| **Citation** | **Q1** | **Q2** | **Q3** | **Q4** | **Q5** | **Q6** | **Q7** | **Q8** | **Q9** | **Q10** |
|---|---|---|---|---|---|---|---|---|---|---|
| Choquette, Rennick and Lee[12] | Y | Y | Y | Y | Y | N | N | Y | Y | Y |
| Walker et al.[44] | N | N | N | N | N | N | N | Y | Y | Y |
| Chen et al.[10] | N | Y | Y | Y | Y | Y | Y | Y | Y | Y |
| Suntup[40] | N | N | N | N | N | N | N | N | Y | Y |
| Vancer, Eiser and Horne[42] | Y | Y | Y | Y | Y | Y | N | Y | Y | Y |
| Vanclooster et al.[43] | N | N | N | N | N | U | Y | Y | Y | Y |
| % | 33.33 | 50.0 | 50.0 | 50.0 | 50.0 | 33.33 | 33.33 | 83.33 | 100.0 | 100.0 |

**Table.** Critical quality appraisal
