# Supplementary material for: “Chemobrain” in childhood cancer survivors—the impact on social, academic, and daily living skills: a qualitative systematic review
Source: Support Care Cancer. 2023 Aug 22;31(9):532. doi: 10.1007/s00520-023-07985-z (PMC10444646; doi:10.1007/s00520-023-07985-z)
Supplement: Supplementary file 6 — Supplementary file6 (PDF 146 KB) [file 520_2023_7985_MOESM6_ESM.pdf]

“Chemobrain” in childhood cancer survivors – the impact on social, academic, and daily living skills: a qualitative systematic review

Ines Semendric[1*], Danielle Pollock[2], Olivia J Haller[1], Rebecca P George[1], Lyndsey E. Collins-Praino[1], Alexandra Whittaker[3]

1. School of Biomedicine, The University of Adelaide, Adelaide, South Australia

2. JBI, Faculty of Health and Medical Sciences, Adelaide, South Australia

3. School of Animal and Veterinary Sciences, The University of Adelaide, Roseworthy, South Australia

*Corresponding author: Ines Semendric

Email: ines.semendric@adelaide.edu.au

Online Resource 6: List of Study Findings with illustrations

Study: Choquette 2016	
Finding	Fatigue, concentration, and memory problems (U)
Illustration	"It's just the mental stuff. Like that's the main part... It's just...I'm slow." p.399
Study: Walker 2019	
Finding	Feeling well mentally and physically (U)
Illustration	"I just feel like a normal student." p.16
Finding	Discerning whether a behavior was related to "chemo brain" or "just being a teenager" (U)
Illustration	“Um, maybe I guess with my laundry, um, as an example like sometimes I don’t wanna do it just "cause my mom like keeps telling me to like put

	it away and stuff. And so like the teenage attitude will be like, no I don't wanna do it, I'm not gonna do it, I'm just gonna leave it there on the chair. There are also times where she'll tell me, and I like, and I'll be like okay I'll do it. But then chemo brain happens, and I forget completely all about it. But then it's kind of like, my mom will be like, 'I told you to put that away.' And I'll be like I forgot. And then she's like, 'Did you really forget or did you just not wanna do it?' So right now the, you're all trying to sort it all out." p.17
Finding	Chemo brain affected their ability to get back into school and catch up on school work (U)
Illustration	"Like the chemo brain and stuff this year, it got hard, harder for me to process things in school." p.17
Finding	Apprehension (U)
Illustration	"My parents want me to like, do like running start in junior year and like do honors classes. But I like, I wanna do that too so I can like excel, but I know like I can't push myself too much, but I also like, "cause like the chemo brain kind of isn't, doesn't wanna like work with that.'' p.17

Study: Vance 2004	
Finding	Struggles they had in obtaining the necessary support for their child (U)
Illustration	“We spoke to [the clinical psychologist] and he did an IQ thing and they said she didn’t need it [special education]. But I think that in itself is unfair as well, because she was so bright before she got poorly” p.276
Finding	Developed other ways of excelling at school (U)
Illustration	“She’s incredibly artistic […] she makes lovely cards, and even just things like brooches and things out of plaster of paris, and then she paints them” Rebecca’s father and mother p.277
Finding	Future employability (U)
Illustration	“I mean I think she’s going to need a lot of supervision and mentoring, whatever she does [...] She won’t be employable unless you’ve got a very considerate employer’’ p.277
Finding	Future concerns over their child's ability to independently self-care (U)
Illustration	I am wary about her moving out...I don't think she's ready at the moment...But I think 12 months from now she'll be much more capable of moving out into a place of her own. I want to

	give her the opportunity to have her own money, her own benefits, so she can spend how she wants and learn how to look after money, which she's still not quite experienced at. Also, to have her paying some of the bills here, some of my bills - give her the money and let her pay them. I'm just trying to make sure that she's ready for anything that crops up when she does leave home. So I'm going to have her doing that over the next 12 months, so that she's ready when she does move out (Lynne)" p.283
Finding	Had been 'statemented' (had been giving a statement of special needs) (U)
Illustration	"She was supposed to get a lot of extra help. In a way it's like a special school within a mainstream school, a special unit (Rebecca)." p.276
Finding	Child's level of independence (U)
Illustration	"I've been really worried about leaving her in the house, but I've had to, and I have to keep telling myself that she's 15 now and that sometimes I do have to go out (Rebecca)." p.281
Finding	Develop social phobia as a result of the academic problems (C)

Illustration	"She would genuinely have been feeling sick, because she was frightened of going to school" Hannah's mother, p.276
Study: Chen 2015	
Finding	Make an effort to live normally (U)
Illustration	"Since his cancer, he had few social interactions and little initiative. However, he has to face his life. He needs to work by himself." p.224
Finding	Learning difficulties and fatigue worsened adolescent's academic performance (U)
Illustration	"I didn't understand what the teachers were teaching [shrugged his shoulders]. So I worried about my poor school performance." p.223
Study: Vanclooster 2021	
Finding	Performance, comparison with peers, and confrontation with physical or educational changes (C)
Illustration	"He knows that he lacks certain skills, now even more than before." (Case 3, Parent) p.2615.
Study: Suntup 1999	
Finding	Behaviors that are indicative of attention difficulties (C)

Illustration	"During the clinical interview with Peter's parents, they reported that Peter displays many behaviors that are indicative of attention difficulties. They noted that Peter fidgets, has difficulty remaining seated, and has difficulty following instructions and sustaining his attention. (...) Peter shifts from one activity to another, does not play quietly, talks excessively, and does not listen. They further noted that he loses things and often engages in physically dangerous activities." (Peter) p.122
Finding	Slow to complete work (C)
Illustration	"When asked about his performance at school, Mrs. B. noted that the report cards never indicated any problem, but that she recognized that he was slow to complete work. Both Mr. and Mrs. B appear to view this slowness as resulting from Peter's tendency to daydream and to become distracted doing his homework, as well as his high activity level. They reported that he does not sit still enough to complete the work." (Mother of Peter) p.113
